# Whole-Genome Resequencing of Seven Eggplant (*Solanum melongena*) and One Wild Relative (*S. incanum*) Accessions Provides New Insights and Breeding Tools for Eggplant Enhancement

**DOI:** 10.3389/fpls.2019.01220

**Published:** 2019-10-08

**Authors:** Pietro Gramazio, Haidong Yan, Tomas Hasing, Santiago Vilanova, Jaime Prohens, Aureliano Bombarely

**Affiliations:** ^1^Faculty of Life and Environmental Sciences, University of Tsukuba, Tsukuba, Japan; ^2^Instituto de Conservación y Mejora de la Agrodiversidad Valenciana, Universitat Politècnica de València, Valencia, Spain; ^3^School of Plant and Environmental Sciences (SPES), Virginia Tech, Blacksburg, VA, United States; ^4^Department of Biosciences, Università degli Studi di Milano, Milan, Italy

**Keywords:** whole-genome resequencing, *Solanum melongena*, *S. incanum*, polymorphisms, genome characterization

## Abstract

Whole-genome resequencing provides information of great relevance for crop genetics, evolution, and breeding. Here, we present the first whole-genome resequencing study using seven eggplant (*Solanum melongena*) and one wild relative (*Solanum incanum*) accessions. These eight accessions were selected for displaying a high phenotypic and genetic diversity and for being the founder parents of an eggplant multiparent advanced generation intercrosses population. By resequencing at an average depth of 19.8× and comparing to the high-quality reference genome “67/3” over 10 million high-reliable polymorphisms were discovered, of which over 9 million (84.5%) were single nucleotide polymorphisms and more than 700,000 (6.5%) InDels. However, while for the *S. melongena* accessions, the variants identified ranged from 0.8 to 1.3 million, over 9 million were detected for the wild *S. incanum*. This confirms the narrow genetic diversity of the domesticated eggplant and suggests that introgression breeding using wild relatives can efficiently contribute to broadening the genetic basis of this crop. Differences were observed among accessions for the enrichment in genes regulating important biological processes. By analyzing the distribution of the variants, we identified the potential footprints of old introgressions from wild relatives that can help to unravel the unclear domestication and breeding history. The comprehensive annotation of these eight genomes and the information provided in this study represents a landmark in eggplant genomics and allows the development of tools for eggplant genetics and breeding.

## Introduction

Common eggplant (*Solanum melongena* L., 2*n* = 2*x* = 24) is the fifth economically most important vegetable crop, and in the last few years, it has undergone a remarkable increase in yield and total production (Faostat, 2017). Despite its importance in many tropical and subtropical areas, eggplant has lagged behind other major crops in the development of genetic and genomic tools (Gramazio et al., 2018). However, recent efforts have been done to close the gap between eggplant and other model species where more genomic information is available (Yang et al., 2014; Gramazio et al., 2016; Zhou et al., 2016). In this way, the significant improvements in next-generation sequencing (NGS) technologies and the associated substantial cost reductions have fostered genomic studies for crops like eggplant. In this respect, the release of reference whole-genome sequences represents an important landmark for undertaking other genomic studies, as it has occurred for the most economically important cultivated crops (Weigel and Mott, 2009; Xu et al., 2012; Aflitos et al., 2014; Zhou et al., 2015; Du et al., 2018).

In eggplant, the first draft of a reference genome was released in 2014, which was built on 33,873 scaffolds and covered 833.1 Mb (∼74% of the eggplant genome) (Hirakawa et al., 2014). A new high-quality eggplant genome sequence from accession “67/3,” assembled combining by Illumina sequencing and optical mapping, has recently been released (http://www.eggplantgenome.org/) (Barchi et al., 2019a). As result, the improved eggplant genome has a lower fragmentation, a higher contiguity, and a higher genome coverage (1.06 of 1.20 Gb estimated genome size) compared with the 2014 version. Together with the draft reference genome of Hirakawa et al. (2014), the “67/3” high-quality reference genome will facilitate resequencing studies in cultivated eggplant and its wild genepool. It will also contribute to accelerate the understanding of the genetic basis of many traits of interest in breeding for which there is a lack of specific molecular and genomic tools. To our knowledge, no resequencing studies have been reported in eggplant, while in model species and other economically important crops, such as *Arabidopsis thaliana* (Cao et al., 2011; Alonso-Blanco et al., 2016), tomato (Aflitos et al., 2014; Lin et al., 2014; Gao et al., 2019), or soybean (Lam et al., 2010; Zhou et al., 2015), dozens, hundreds, and even thousands accessions have been resequenced. Examples include. In fact, in these studies, the resequencing approach has allowed capturing the natural variation across the genepool through the identification of millions of robust polymorphisms among cultivated and wild relative accessions. The high-confidence sets of polymorphisms provided a valuable opportunity to perform forward genetics and genome-wide association studies and unravel the genetic base of complex traits of agronomic importance (Huang et al., 2010; Tranchida-Lombardo et al., 2017). In addition, whole-genome resequencing helps to gain insight on the evolution of crops by identifying genetic diversity bottlenecks that occurred during the domestication, as well as the genes involved in the process (Zhou et al., 2015; Gao et al., 2019). Furthermore, resequencing studies have allowed associating genes and traits with geographical areas revealing how populations and subpopulations have adapted to specific geographical regions (Qi et al., 2013). Ultimately, the whole-genome resequencing approach is a powerful breeding tool that can foster the development of a new generation of resilient varieties adapted to present and future challenges like climate change.

In this paper, we describe the first whole-genome resequencing study in the eggplant genepool including a comprehensive structural and functional characterization of seven *S. melongena* and one wild eggplant relative (*S. incanum* L.) accessions of interest for eggplant breeding (Prohens et al., 2013; Gramazio et al., 2017a). These eight accessions were chosen to maximize the phenotypic, genetic, and geographic variation and are the founder parents of a multiparent advanced generation intercrosses (MAGIC) population that is under development. *Solanum incanum*, which is part of the secondary genepool of the common eggplant (Syfert et al., 2016), has been reported as a powerful source of phenolic compounds, displaying contents several times higher than those of *S. melongena* (Stommel and Whitaker, 2003; Prohens et al., 2013), and is tolerant to some biotic and abiotic stresses, mainly drought (Knapp et al., 2013). The aim of this study was to interrogate the entire genome of these accessions to provide a large set of robust and high-confidence molecular markers in a very diverse eggplant germplasm set for breeding purposes, genetic and genomic studies, and to develop a high-throughput genotyping platform to efficiently assist the future characterization of the MAGIC population under development. In addition, this exploratory study intends to pave the way for future studies to shed light on eggplant evolutionary and breeding history through the characterization of introgressions, transposable, and genomic elements. The information and tools developed in our study will be of great relevance for eggplant genomics-assisted breeding.

## Materials and Methods

### Plant Materials

**Figure 1:**
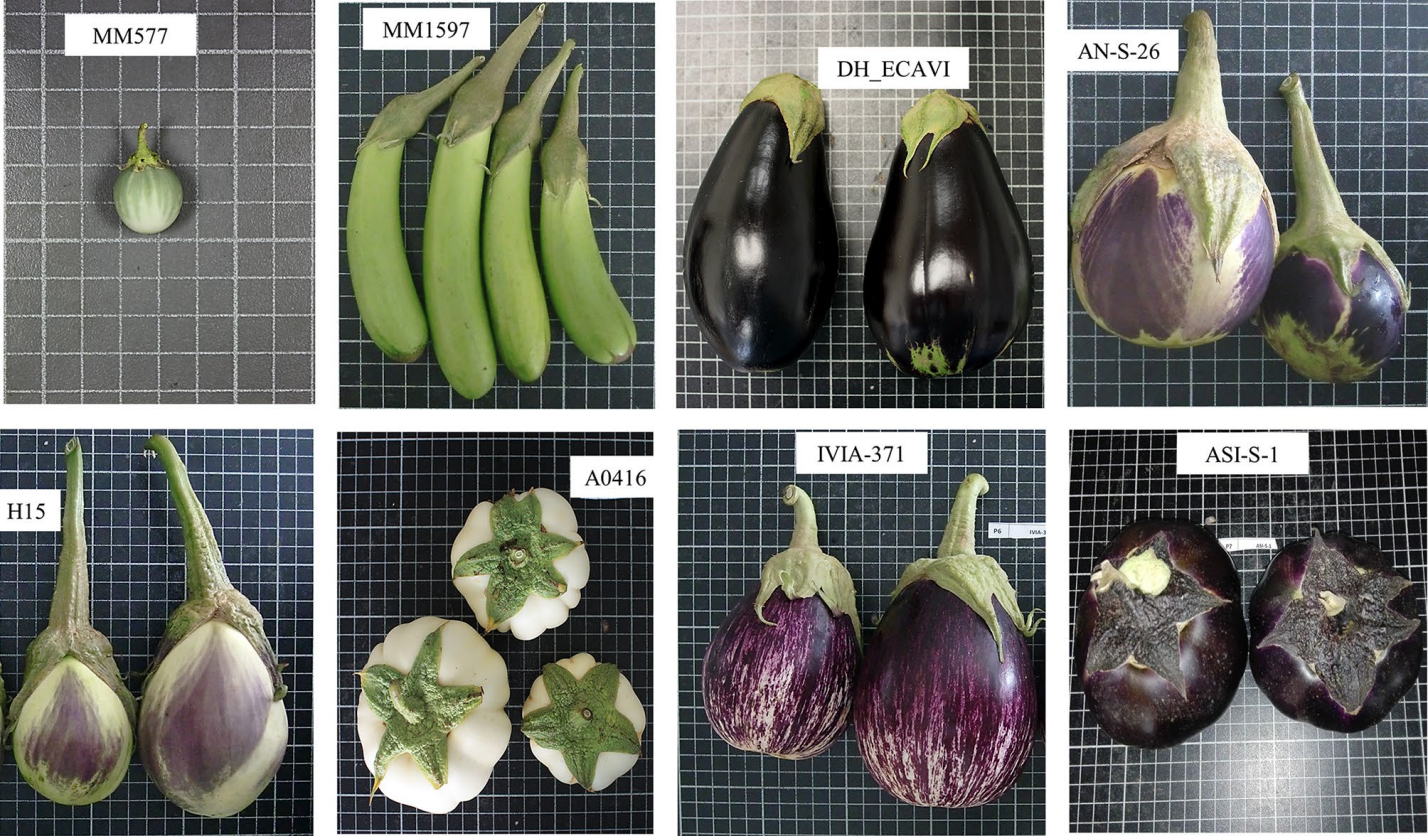
Pictures of the seven *Solanum melongena* and one *S. incanum* (MM577) accessions resequenced in this study. Fruits are not depicted at the same scale; the size of the grid cells is 1 cm × 1 cm.

Seven *Solanum melongena* accessions were chosen to maximize the representation of phenotypic and genetic diversity in the common eggplant (**Table 1**). The accessions have different origins and display substantial differences in agronomic and morphological traits, such as prickliness, fruit size, fruit shape, fruit color, or calyx size (**Figure 1**). In addition, one *S. incanum* accession (MM577) was selected as an outgroup because of its interest in eggplant breeding. This specific *S. incanum* accession has been characterized in multiple genetic and genomic studies for several traits (Stommel and Whitaker, 2003; Gisbert et al., 2011; Salas et al., 2011; Prohens et al., 2013; Meyer et al., 2015; Gramazio et al., 2016; Gramazio et al., 2017b) and has been used as a parent for developing an interspecific linkage genetic map and the first introgression line population in the eggplant genepool (Gramazio et al., 2014, Gramazio et al., 2017a). All the accessions are maintained at the Universitat Politècnica de València (UPV) germplasm bank. Seeds were germinated in Petri dishes, following the Ranil et al. (2015) protocol, and were subsequently transferred to seedling trays in a climatic chamber under a photoperiod and temperature regime of 16 h light (25°C)/8 h dark (18°C). The plantlets were then transplanted to a glasshouse situated in the campus of the UPV, Valencia, Spain (GPS coordinates: latitude, 39° 28′ 55″ N; longitude, 0° 20′ 11″ W; 7 m above sea level). Plants were grown in 15-L pots filled with coconut fiber, irrigated and fertilized using a drip irrigation system, and pruned and trained with vertical strings.

**Table 1:** Plant materials used in this study including their country of origin and main phenotypic characteristics.

Taxon and accession	Country of origin	Fruit shape^a^	Fruit size^b^	Predominant fruit color^c^	*PUC* allele^d^	Fruit stripes
*S. incanum* L.						
MM577	Israel	3	1	1.2	Unknown^e^	Yes
*S. melongena* L.						
MM1597	India	9	7	1.2	Unknown^e^	No
DH_ECAVI	Breeding line	7	7	8	Yes	No
H15	Spain	5	3	7	No	No
AN-S-26	Spain	5	5	7	No	No
A0416	Unknown	1	5	2	Unknown^e^	No
IVIA-371	Spain	5	9	7	Yes	Yes
ASI-S-1	China	1	5	8	No	No

a Fruit shape according to the following scale: 1 = broader than long; 3 = as long as broad; 5 = slightly longer than broad; 7 = twice as long as broad; 9 = several times as long as broad.

b Fruit size according to the following scale: 1 = very small; 3 = small; 5 = medium; 7 = large; 9 = very large.

c Fruit predominant color when the fruit is physiologically immature according to the following scale: 1 = green; 3 = white; 5 = clear purple; 7 = purple; 9 = purple black.

d Presence of PUC [pigment (anthocyanins) under calyx] dominant allele.

e The fruit skin does not contain anthocyanins, and therefore, the PUC allele presence is unknown.

### Sequencing

Total genomic DNA was isolated from an individual plantlet of each accession according to the cetyl trimethylammonium bromide method (Doyle and Doyle, 1990), with slight modifications. DNA integrity was evaluated with agarose electrophoresis, DNA quality through the 260/280 and 260/230 nm ratios from NanoDrop ND-1000 spectrophotometer (NanoDrop Technologies, Wilmington, Delaware, USA) and concentration with a Qubit^®^ 2.0 Fluorometer (Thermo Fisher Scientific, Waltham, MA, USA). High-quality DNA samples were shipped to the Duke Center for Genomic and Computational Biology (Durham, NC, USA) for libraries construction and sequencing. Paired-end libraries were prepared using the KAPA HyperPrep Kits (Roche, Basel, Switzerland) with an insert size of approximately 300 bp and sequenced on two lines of an Illumina HiSeq 4000 sequencer.

### Sequence Processing, Mapping, and Polymorphisms Calling

The raw sequences were processed with the Ea-utils package for barcode demultiplexing, adapter trimming, quality filtering, and stats (Aronesty, 2013). Raw reads shorter than 50 bp and with Phred score lower than 30 were removed with the fastq-mcf tool. The processed reads were then mapped to the “67/3” high-quality eggplant reference genome (Barchi et al., 2019a; http://www.eggplantgenome.org/) with the Bowtie2 aligner (version 2.2.4), which was selected for its speed and capability to give a lower score for suboptimal alignments (Li, 2014), using default parameters (Langmead and Salzberg, 2012). The unmapped reads were assembled with the short-read assembler Minia with default settings (Salikhov et al., 2013), and the contigs obtained were blasted against the nr database of the GenBank. The SAMtools package (version 1.3.1) was used to convert the mapping results into the BAM format and to filter the unmapped reads (Li et al., 2009). The coverage of sequence alignments was calculated using the utility genomecov of the BEDtools package (version 2.21.0; Quinlan and Hall, 2010), while the average coverage was determined with an in-house script. Variant calling was performed using the Bayesian-based FreeBayes software (v0.9.20, Garrison and Marth, 2012) with a minimum depth coverage of 5, a minimum base quality of 20, and a minimum mapping quality of 20. The vcftools package (version 0.1.12; Danecek et al., 2010) was used to split the main variant call format (VCF) output file into eight individual files and to remove the allele missing data. In order to identify similar patterns of polymorphisms distribution to associate them with potential common ancestral introgressions, the main VCF file was divided into homozygous (0/0) or heterozygous (0/1) variants and into segments of 10 Mb. The number of variants for each segment was calculated with the utility Vcf2CountingBins of the package GenoToolBox (https://github.com/aubombarely/GenoToolBox) and plotted by chromosomes in R (Ihaka and Gentleman, 1996). Using the whole set of single nucleotide polymorphisms (SNPs), after filtering out the other marker types [InDels, multiple-nucleotide polymorphisms (MNP), and complex] and the missing data, a genetic matrix distance was calculated among the different accessions using the TASSEL software (version 5.0 Standalone, Bradbury et al., 2007). A multivariate principal coordinates analysis (PCoA) was performed using the “prcomp” (https://stat.ethz.ch/R-manual/R-devel/library/stats/html/prcomp.html) and “factoextra” (https://cran.r-project.org/web/packages/factoextra/index.html) packages of the R software.

### Variants and Genome Structural and Functional Annotation

The variants annotation was performed according to the “67/3” eggplant reference genome gff3 annotation file using the Bedtools package (version 2.21.0; Quinlan and Hall, 2010) and Blast2GO software (Conesa et al., 2005). In addition, the Gene Ontology (GO) terms were added from the GO database (http://www.geneontology.org/). The variant effects were annotated based on their genomic position using the SnpEff software (version 4.2; Cingolani et al., 2012). Specifically, the effects of the allelic variants were classified by impact (high, low, moderate, or modifier), by functional class (missense, nonsense, or silent mutation), by type (start lost, stop gained, stop lost, and others), and region affected (intergenic, intron, exon, and others). In addition, the DNA substitution mutations (transitions and transversion) and amino acids changes were reported.

### Transposon Comparison

Transposons in the eggplant reference genome “67/3” were searched by combining a *de novo* and a homology-based approaches. For the *de novo*-based approach, the RepeatModeler software (Smit and Hubley, 2015) was used for identifying repeat element boundaries and family relationships from the reference genome sequence. For the homology-based approach, RepeatMasker program (Smit et al., 2004) was used to obtain a detailed annotation of the repeats from the screening for interspersed repeats and low complexity DNA sequences against the Repbase TE library (https://www.girinst.org/). Subsequently, the transposons found with the two approaches were combined, and the redundant hits were removed using CD-HIT software (Fu et al., 2012) with default parameters. Transposon insertions were detected with the TEMP software (Zhuang et al., 2014), and their quantity were calculated based on the output of the McClintock pipeline (Nelson et al., 2017). Transposon size was calculated based on bed files generated from the McClintock pipeline.

### Data Access

The sequence data have been deposited into NCBI Short Read Archive under submission identifier SUB2829594 with the Bioproject identifier PRJNA392603. Raw reads of each accession are deposited under the accession numbers from SRR5796636 to SRR5796643. VCF files with the corresponding variants identified are available upon request to the corresponding author.

## Results

### Whole-Genome Resequencing and Mapping

The genome sequencing of the eight accessions generated over 1.4 billion paired-end raw reads (109 Gb of data), with a mean of 180.9 million reads per sample (**Table 2**). After the cleaning step, 97.6% of sequences, with an average length of 135 bp, were kept and were mapped onto the eggplant reference genome “67/3” (Barchi et al., 2019a). The mapping rate was quite similar across the accessions with an average of 85.4% and a range from 76.9% (ASI-S-1) to 88.7% (MM1597). The lower mapping percentage of the *S. melongena* accession ASI-S-1 was due to a higher proportion of mitochondrial reads that did not map to the reference genome since the full mitochondrial genome is not included in the reference genome “67/3.” The mean coverage depth varied from 16.5x in H15 to 24.0x in A0416, with a mean in the set of accessions of 19.8x. The mapping coverage of common eggplant (*S. melongena*) accessions encompassed practically the whole length of the reference genome, except for small regions (∼1%) of chromosomes 1, 11, and 12 in A0416, chromosomes 9 and 12 in AN-S-26, and chromosome 12 in DH_ECAVI, H15, and IVIA-371 (**Supplementary Data S1**). The *S. incanum* mapping coverage was lower, with a mean of 95.4%, and a quite even distribution along the chromosomes.

**Table 2:** Statistics of sequencing and mapping of the seven *Solanum melongena* and one *S. incanum* accessions resequenced in this study.

	*S. incanum*	*S. melongena*							Mean	Total
	MM577	MM1597	DH_ECAVI	AN-S-26	H15	A0416	IVIA-371	ASI-S-1		
*Raw reads (million)*	151.4	173.5	199.1	182.1	153.1	218.1	195.0	175.4	180.9	1,448.00
*High-quality reads (million)*	149.0	170.7	194.2	179.1	148.8	214.1	190.2	172.1	177.3	1,418.60
*% cleaning after cleaning*	98.4	98.3	97.5	98.3	97.2	98.1	97.5	98.1	97.6	–
*Nucleotides after cleaning (billion)*	44.0	50.5	57.6	53.0	44.2	63.3	56.2	50.3	52.4	419.3
*Reads mapped (million)*	120.5	151.5	171.1	158.5	130.3	188.6	162.7	132.4	152.0	1,216.00
*% reads mapped*	80.8	88.7	88.1	88.4	87.5	88.0	85.5	76.9	85.4	–
*Mean depth after mapping*	16.6	19.5	21.5	20.6	16.5	24.0	19.9	19.9	19.8	–
*% coverage reference genome*	95.4	∼100.0	∼100.0	∼100.0	∼100.0	∼100.0	∼100.0	∼100.0	99.4	–

### Genomic Variations and Distribution

A total of 12,243,422 polymorphisms were detected among the eight resequenced accessions). To retain reliable variants for genotyping purposes and population studies, missing data were excluded when individual accessions were compared to the reference genome, yielding a final total of 10,916,466 high-quality variants (**Table 3**). Of those, 9,228,065 were SNPs (84.5%), 705,687 InDels (6.5%), 275,467 MNPs (2.5%), and 707,247 complex variations (6.5%). The total number of polymorphisms was homogeneously distributed among the *S. melongena* accessions and ranged from 832,366 of ASI-S-1 to 1,282,595 of IVIA-371. As expected, the wild *S. incanum* presented a much higher number of polymorphisms, with 9,343,703 variants when compared to the eggplant reference genome “67/3”.

**Table 3:** Summary of the polymorphisms identified in the seven *S. melongena* and one *S. incanum* accessions of this study using the *S. melongena* accession 67/3 as a reference genome (Barchi et al., 2019a).

	*S. incanum*		*S. melongena*															
	MM577		MM1597		DH_ECAVI		AN-5-26		H15		A0416		IVIA-371		ASI-S-1		Total	
	Count	%	Count	%	Count	%	Count	%	Count	%	Count	%	Count	%	Count	%	Count	%
**Variants**																		
*Homozygous SNPs*	3,101,638	33.2	664,586	53.9	656,892	53.4	664,128	54.9	639,408	55.4	691,137	54.9	750,967	58.6	190,118	22.8	9,228,065	84.5
*Heterozygous SNPs*	4,768,817	51.0	372,002	30.2	389,310	31.7	365,676	30.2	345,148	29.9	366,485	29.1	346,213	27.0	529,784	63.6		
*Homozygous InDels*	184,815	2.0	58,108	4.7	55,070	4.5	55,606	4.6	53,708	4.7	58,567	4.6	59,542	4.6	16,347	2.0	705,687	6.5
*Heterozygous InDels*	403,965	4.3	53,242	4.3	50,463	4.1	49,925	4.1	45,520	3.9	53,503	4.2	47,897	3.7	42,866	5.1		
*Homozygous MNP*	72,886	0.8	13,305	1.1	12,620	1.0	12,034	1.0	11,412	1.0	14,959	1.2	13,496	1.1	4,300	0.5	275,467	2.5
*Heterozygous MNP*	179,429	1.9	9,526	0.8	8,938	0.7	8,346	0.7	7,652	0.7	9,251	0.7	8,126	0.6	10,050	1.2		
*Homozygous Complex*	172,805	1.8	34,371	2.8	30,961	2.5	29,716	2.5	28,068	2.4	39,017	3.1	32,873	2.6	11,253	1.4	707,247	6.5
*Heterozygous Complex*	459,348	4.9	27,343	2.2	25,335	2.1	24,013	2.0	22,352	1.9	26,780	2.1	23,481	1.8	27,648	3.3		
*Total homozygous*	3,532,144	37.8	770,370	62.5	755,543	61.4	761,484	63.0	732,596	63.5	803,680	63.8	856,878	66.8	222,018	26.7	–	–
*Total heterozygous*	5,811,559	62.2	462,113	37.5	474,046	38.6	447,960	37.0	420,672	36.5	456,019	36.2	425,717	33.2	610,348	73.3	–	–
*Total variants*	9,343,703	100.0	1,232,483	100.0	1,229,589	100.0	1,209,444	100.0	1,153,268	100.0	1,259,699	100.0	1,282,595	100.0	832,366	100.0	10,916,466	100.0
*Private variants*	7,949,491	85.0	179,876	14.5	59,353	4.8	80,661	6.6	83,494	7.2	217,808	17.2	73,851	5.7	99,617	11.9	8,744,151	80.1
*% variants with the reference genome*	0.8177		0.1079		0.1076		0.1058		0.1009		0.1102		0.1122		0.0728		–	
**Variants in coding regions**																		
*Homozygous SNPs*	277,313	42.9	58,736	54.6	52,564	49.9	51,368	51.2	49,347	48.8	59,048	55.0	54,995	52.4	14,215	24.0	697,205	81.7
*Heterozygous SNPs*	246,123	38.1	26,392	24.5	30,868	29.3	27,810	27.7	31,031	30.7	25,152	23.4	28,392	27.0	33,623	56.9		
*Homozygous InDels*	31,501	4.9	8,844	8.2	8,299	7.9	8,244	8.2	7,983	7.9	9,137	8.5	8,522	8.1	2,176	3.7	102,725	12.0
*Heterozygous InDels*	52,452	8.1	7,598	7.1	7,563	7.2	7,334	7.3	7,131	7.1	7,662	7.1	7,224	6.9	5,713	9.7		
*Homozygous MNP*	4,470	0.7	881	0.8	815	0.8	790.0	0.8	737	0.7	967	0.9	840	0.8	268	0.5	14,535	1.7
*Heterozygous MNP*	6,801	1.1	766.0	0.7	818	0.8	718.0	0.7	755	0.7	744.0	0.7	778	0.7	647	1.1		
*Homozygous Complex*	9,187	1.4	2,129	2.0	1,893	1.8	1,889	1.9	1,764	1.7	2,344	2.2	1,977	1.9	734	1.2	39,176	4.6
*Heterozygous Complex*	18,244	2.8	2,220	2.1	2,423	2.3	2,249	2.2	2,376	2.3	2,275	2.1	2,323	2.2	1,765	3.0		
*Total homozygous*	322,471	49.9	70,590	65.6	63,571	60.4	62,291	62.0	59,831	59.2	71,496	66.6	66,334	63.1	17,393	29.4	–	–
*Total heterozygous*	323,620	50.1	36,976	34.4	41,672	39.6	38,111	38.0	41,293	40.8	35,833	33.4	38,717	36.9	41,748	70.6	–	–
*Total variants*	646,091	100.0	107,566	100.0	105,243	100.0	100,402	100.0	101,124	100.0	107,329	100.0	105,051	100.0	59,141	100.0	853,641	100.0

In all accessions, the total number of homozygous variants was higher (above 60%) than the heterozygous ones, except in the common eggplant ASI-S-1 and in the wild *S. incanum* MM577, where the total heterozygous variants represented 73.3% and 62.2% of the total variants, respectively, which may be due to the different degree of fixation among the eight accessions. The number of private variants (i.e., accession specific) was variable among the common eggplant accessions and ranged from 4.8% in DH_ECAVI with 59,353 polymorphisms to 17.2% in A0416 with 217,808 variants, while in the *S. incanum* MM577 accession, the percentage rose to 85.1% with over 8.7 million private variants (**Table 3**). Considering the variants between the accessions and the reference genome and its total length in base pairs, the percentages of variants with respect to the reference genome sequence ranged between 0.072% of ASI-S-1 and 0.112% of IVIA-371 in common eggplant and reached 0.817% with *S. incanum* accession MM577.

The variants identified in coding regions were much less abundant (853,641 and 7.8% of the total). Of these, 81.7% were SNPs, 12.0% InDels, 1.7% MNPs, and 4.6% complex variants. When considering only the seven *S. melongena* accessions, the total variants ranged from 59,141 in ASI-S-1 to 107,566 in MM1597, while in the *S. incanum* accession, there were 646,091 polymorphisms in coding regions. However, the gap in variants between the common eggplant accessions and the wild *S. incanum* decreased from almost 8-fold in the whole genome to around 6.5-fold in coding regions. Interestingly, while the rest of accessions maintained approximately their ratios of homozygous/heterozygous variants in the coding regions (from 71.4% in A0416 to 59.2% homozygous variants in H15), the *S. incanum* MM577 accession showed an almost equal proportion of them (322,471 homozygous vs. 323,620 heterozygous variants).

Substantial differences were observed in the average number of polymorphisms among the chromosomes, with differences over 4-fold between chromosome 5 (43,098) and chromosome 12 (181,603) among the seven common eggplant accessions and over 4.5-fold between chromosome 9 (257,781) and chromosome 7 (1,195,706) in the *S. incanum* accession (**Supplementary Data S2**). In addition, considerable variation within individual chromosome for the total number of polymorphisms was found among *S. melongena* accessions. In fact, in chromosome 12, the differences among the *S. melongena* lines was of up to fourfold; in chromosomes 1 and 2, it was around threefold; and in chromosomes 6, 7, and 10, over twofold. However, the most remarkable difference in concentration of polymorphisms was found in chromosome 1 for A0416 with 314,756 variants, while the mean for the rest of the lines was 107,979. The variants rate (physical length divided by the number of variants) was quite uniform for all the common eggplant accessions (on average 1 variant every 976 bp), ranging from one variant every 891 bp in IVIA-371 to one variant every 1,373 bp in ASI-S-1. For the *S. incanum* accession, the variation rate was considerably higher than for the *S. melongena* accessions (one variant every 122 bp), although for this calculation, we used the length of the *S. melongena* reference genome since the exact physical length of the *S. incanum* genome is still unknown. Regarding the average variation rate per chromosomes in the *S. melongena* accessions, the lowest value was found in chromosome 4 (one variant every 1,511 bp) and the highest in chromosome 12 (one variant every 553 bp). The range of variation for the *S. incanum* accession MM577 was narrower; chromosome 9 had the lowest rate with one variant every 140 bp, while chromosomes 2 and 8 had the highest rate with one variant every 117 bp. The number of variants was highly related to the physical length of the chromosomes since, for common eggplant accessions, the correlation was *r* = 0.7012 (*p* = 0.01105) and for *S. incanum r* = 0.9946 (*p* = 3.433 × 10^-11^).

**Figure 2:**
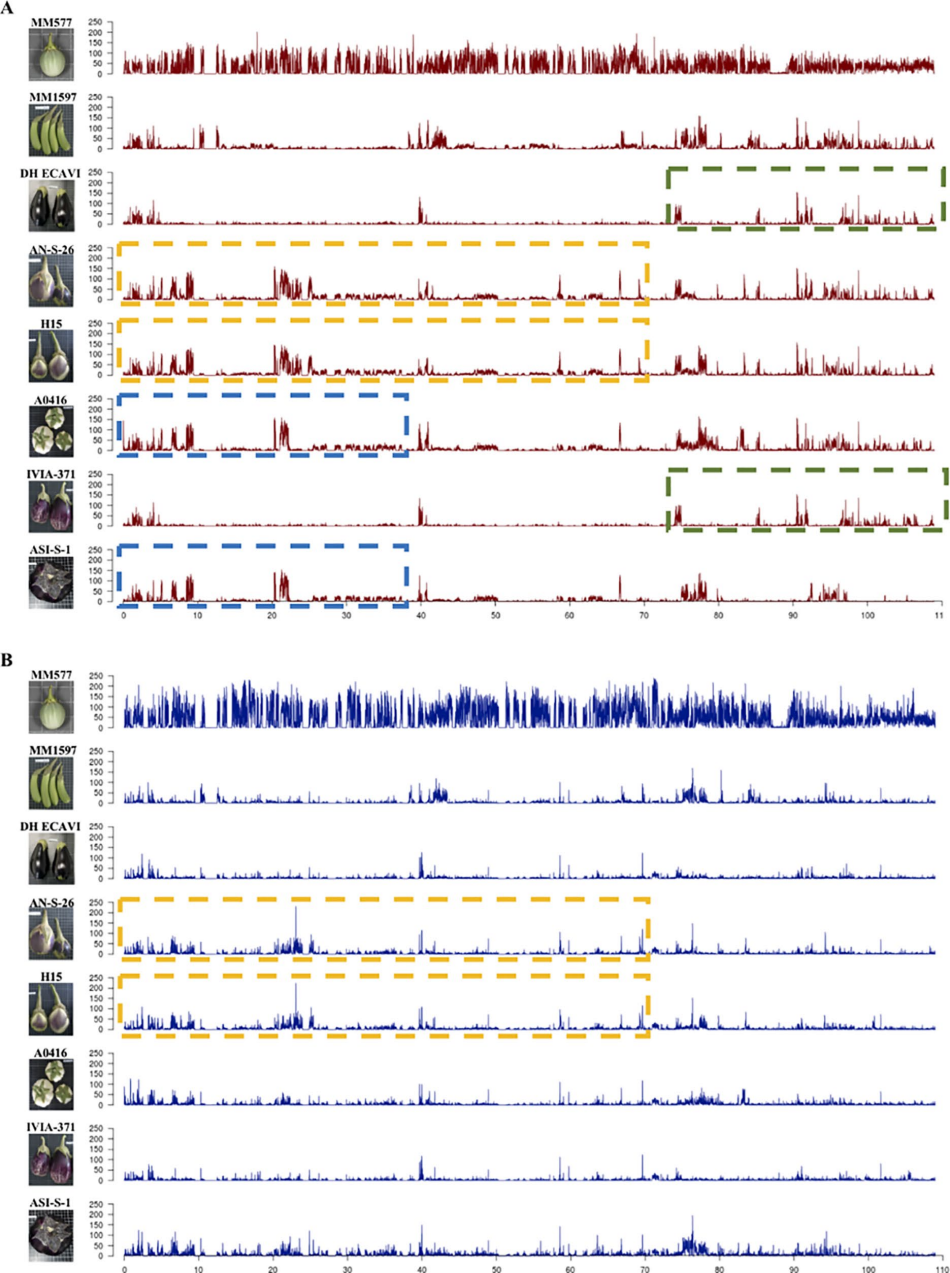
Distribution of homozygous **(A)** and heterozygous **(B)** polymorphisms along chromosome 6 for the seven *S. melongena* and one *S. incanum* (MM577) accessions resequenced identified using the *S. melongena* accession 67/3 as a reference genome (Barchi et al., 2019a). The peaks represent high frequencies of polymorphisms in a window size of 10 Mbp. The dashed lines of the same color indicate similar patterns of polymorphisms distribution.

The variants were separated in heterozygous and homozygous, and their distribution along the chromosomes was plotted (**Figure 2**; **Supplementary Data S3**). The peaks represent high concentrations of variants in a window size of 10 Mbp. Overall, the heterozygous variants were distributed more evenly than the homozygous ones, which were more abundant in distal parts compared to centromeric regions. Some accessions showed large regions of almost identical peaks pattern distribution for homozygous variants like DH_ECAVI and IVIA-371 in chromosomes 2, 6, and 11; H15 and IVIA-371 in chromosome 2; AN-S-26 and H15 and A0416 and ASI-S-1 in chromosome 6; AN-S-26, H15, and IVIA-371 in chromosome 9; and DH_ECAVI, AN-S-26, H15, and IVIA-371 in chromosome 12. For heterozygous variants, similar peak patterns were observed for DH_ECAVI and IVIA-371 in chromosomes 2 and 11; AN-S-26 and H15 in chromosome 6; AN-S-26, H15, and IVIA-371 in chromosome 9; and DH_ECAVI, AN-S-26, H15, and IVIA-371 in chromosome 12.

### Variants Annotation

According to the variant effect prediction software SnpEff, 98.72% of the variants were classified as “modifier” (i.e., the variants were located in intergenic or intronic regions, or affected noncoding genes), indicating that there is no evidence of impact or that their predictions are difficult to assess (**Supplementary Data S4**). The second most abundant variants impact class was “moderate” (0.77%) and ranged from 0.61% in MM577 to 0.78% in H15. These are nondisruptive variants, such as codon insertion/deletion or codon substitution, that might change protein effectiveness. The variant with “low” impact effects are those unlikely to change protein behavior, like start or stop codon mutated into another start or stop codon or into the same amino acid, represented an average of 0.46%, ranging from 0.43% in ASI-S-1 to 0.48% in H15. Finally, the less abundant impact class corresponded to the “high” variation effects (0.07%), which are considered to have a disruptive impact on the protein (like truncation or loss of function caused by exon deletion/deletion, start/stop codon mutation, or splice site modification), ranged from 0.05% in *S. incanum* MM577 to 0.09% from the total number of variants of each accession.

Among the variants that had been predicted to cause a high impact effect, the most abundant was the “frameshift variant,” caused by insertions or deletions that disrupt the translational reading frame, with a total mean of 0.037%. The other most abundant variants with high impact were “stop gained,” resulting in a premature stop codon and leading to a shortened polypeptide, with a mean of 0.024%; “stop lost,” corresponding to an elongated transcript due to the loss of stop codon, with a mean of 0.006%; “splice donor variant” and “splice acceptor variant,” causing the change of the two base pair regions at the 5′ end and at the 3′ of an intron, respectively, with total means of 0.006% and 0.005%; and finally, “start lost,” when a variant causes the mutation of a start codon into a nonstart codon, with a mean of 0.005%. Regarding the moderate impact, the “missense variant,” when a change in one or more bases causes a different amino acid sequence, was by far the most abundant effect with 0.717%, followed by “inframe insertion” (0.009%) and “inframe deletion” (0.007%) when one or many codons are inserted or deleted, respectively, and finally, “disruptive inframe insertion” (0.005%) and “disruptive inframe deletion” (0.005%) effects, which occur when one codon is changed and one or more codons are inserted or deleted. For the low impact effects, most polymorphisms were predicted as “synonymous variant” (0.377%), i.e., when a variant causes a codon that produces the same amino acid, followed by “splice region variant” (0.100%) when a variant occurs within the region of the splice site, and “5′ untranslated region (UTR) premature start codon gain variant” (0.012%) when a variant in 5′ UTR region produces a start codon. The modifier impact effects and the classification of variants effects by region indicated that over 93% of the variants were located outside the genes, in intergenic regions (68.4%), upstream (13.2%), and downstream (12.1%) of the genes, followed by intron (4.5%) and exon (1.1%), 3′ UTR (0.24%) and 5′ UTR (0.15%), splice site region (0.081%), transcript (0.03%), splice site donor (0.005%), and acceptor (0.005%). The details of amino acid changes are reported in **Supplementary Data S5**.

*S. incanum* had four times more genes affected by “high” impact effect variations (4,283) than other accessions (from 786 for ASI-S-1 to 1,130 for H15). An analysis of the biological processes (BP) GO enrichment associated to these genes showed a general enrichment in genes associated to the photosynthetic process. For example, “photosynthetic electron transport” (GO:0009772, GO:0009767), “photosynthesis” (GO:0015979), “carbon fixation” (GO:0015977), and/or “ATP synthesis coupled proton transport” (GO:0015986) were enriched for all the accessions (**Supplementary Data S6**). These genes were usually annotated as the different elements of the photosynthetic systems (e.g., SMEL_003g176830.1, “photosystem II D2 protein;” SMEL_002g157390.1, “ATP synthase subunit alpha, chloroplastic;” SMEL_002g159210.1, “Ycf2;” SMEL_005g233370.1, “ribulose bisphosphate carboxylase large chain”) (**Supplementary Data 7**). Nevertheless, there were some differences between accessions. *S. incanum* MM577 showed an enrichment in “telomere maintenance” (GO:0000723) and “DNA repair” (GO:0006281) (mostly helicases-like proteins) and RNA processing as “mRNA cleavage” (GO:0006379), “rRNA modification” (GO:0000154), “ncRNA processing” (GO:0034470), “RNA methylation” (GO:0001510), and “prolyl-tRNA aminoacylation” (GO:0006433) associated to genes such as RNA polymerases (e.g., SMEL_005g226240.1), ribonucleases (e.g., SMEL_009g322130.1), and tRNA-methyltransferases (e.g., SMEL_006g270090.1). In addition, *S. incanum* presented enrichment in genes associated to metabolic pathways such as “GDP-mannose biosynthetic process” (GO:0009298) (e.g., mannose-6-phosphate isomerase SMEL_006g255960.1) and “glutamine family amino acid biosynthetic process” (GO:0009084) (e.g., glutamine synthetase, SMEL_003g183980.1). Accession *S. melongena* MM1597 presented a differential enrichment in “nitrate transport” (GO:0015706) and “response to nitrate” (GO:0010167) associated to the gene SMEL_003g189360.1 (high-affinity nitrate transporter). DH_ECAVI presented a differential enrichment in “TOR signaling” (GO:0031929) linked to the genes SMEL_003g174880.1 and SMEL_010g353100.1 (regulatory-associated protein of TOR 1). Accession H15 had associated the enriched term “leaf formation” (GO:0010338) (gene SMEL_009g323460.1, an MYB transcription factor) and *S. melongena* A0416 genes associated to the term “recognition of pollen” (GO:0048544) (receptor-like protein kinases genes SMEL_001g143850.1, SMEL_004g218090.1, SMEL_006g251240.1, SMEL_007g292990.1, SMEL_007g293020.1, SMEL_007g293120.1, and SMEL_009g324070.1). Finally, *S. melongena* ASI-S-1 showed enrichment in the terms “cell volume homeostasis” (GO:0006884) and “chloride transport” (GO:0006821) associated to the genes SMEL_003g181350.1 (methylosome subunit pICln) and SMEL_009g330700.1 (unknown function).

### Relationship Among the Accessions

**Figure 3:**
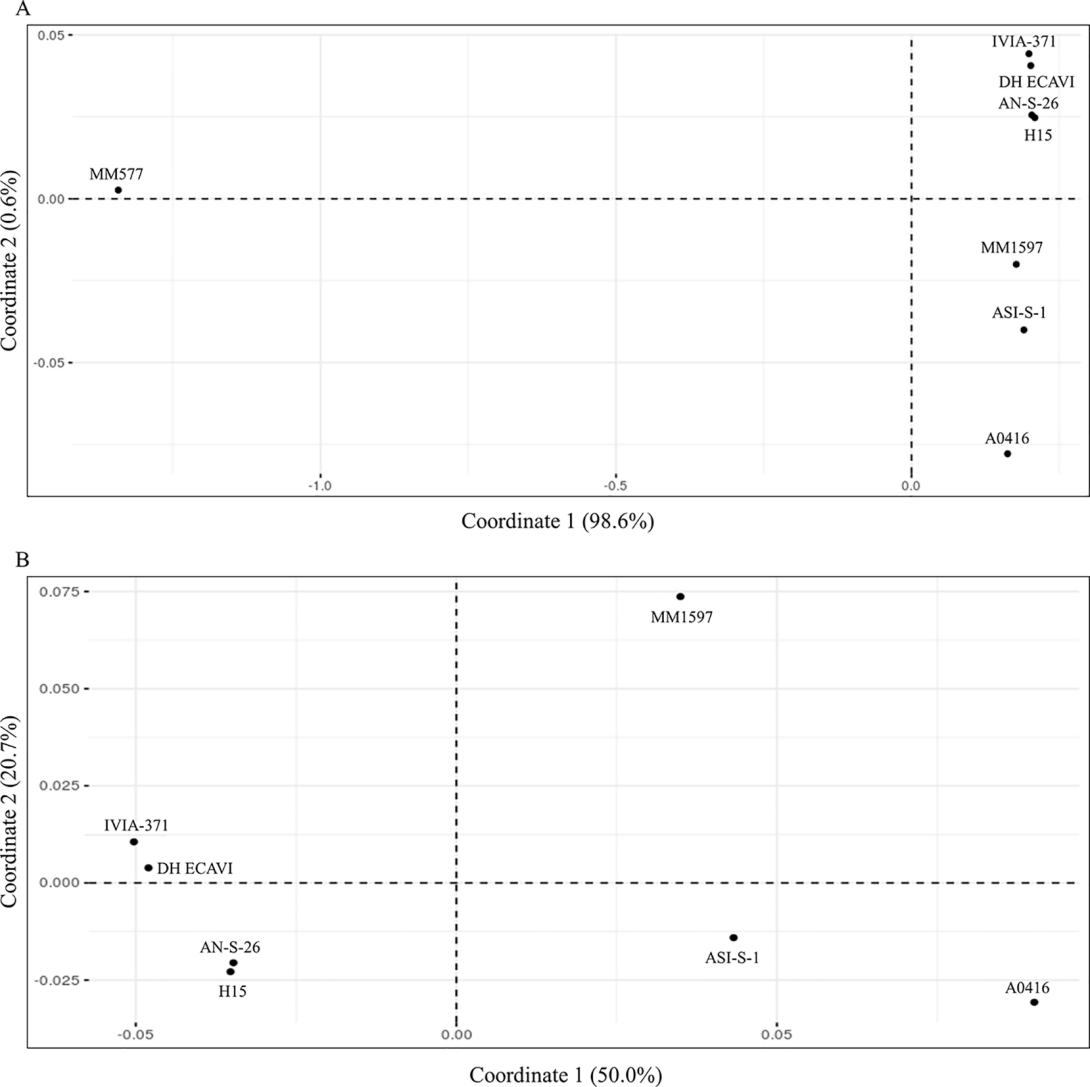
Principal coordinates analysis (PCoA) similarities based on the whole set of SNPs (9,228,065) for the seven *S. melongena* and one *S. incanum* (MM577) accessions **(A)** and on partitioned data without *S. incanum* (MM577) **(B)**. The first and second principal coordinates are displayed.

Genetic identities among the accessions were calculated on 9,109,331 SNPs after removing all the missing data (**Supplementary Data S8**). High values of genetic identities (over 0.9) were obtained among common eggplant accessions, while lower values (below 0.5) were obtained when *S. melongena* accessions were compared with the *S. incanum* MM577. The higher genetic identity values were found between H15 and AN-S-26 (0.965) and between IVIA-371 and DH_ECAVI (0.963). Anyway, the pairwise comparisons of these four varieties resulted in the highest genetic identity values observed (0.951 between H15 and DH_ECAVI, 0.948 between AN-S-26 and DH_ECAVI, 0.947 between H15 and IVIA-371, and 0.946 between IVIA-371 and AN-S-26). Slightly lower genetic identity values were observed between ASI-S-1 and the cluster formed by H15 and AN-S-26 (0.938 and 0.936, respectively), A0416 (0.936), MM1597 (0.929), and the other cluster formed by DH_ECAVI (0.927) and IVIA-371 (0.921). Lower values, even though still quite high (the lowest value being 0.905), were obtained when comparing MM1597 and A0416 with the rest of accessions. As expected, the *S. incanum* accession displayed by far the lowest genetic identity values with a mean of 0.413. These values were also reflected in the multivariate PCoA analysis using the genetic matrix calculated by the TASSEL software (**Figure 3**). The first principal coordinate, which accounted for 98.6% of the variation, clearly separated the common eggplant accessions from the *S. incanum* accession (**Figure 3A**), while the second coordinate, which accounted for 0.6% of the variation, separated the Occidental (DH_ECAVI, AN-S-26, H15, and IVIA-371) from the Oriental accessions (MM1597, ASI-S-1) plus A0416 (of unknown origin). In order to gain a better landscape of the genetic relationships among the *S. melongena* accessions, a further analysis on partitioned data was performed by removing *S. incanum* (**Figure 3B**). The first axis, accounting for 50.0% of the intraspecific genetic variation, separated the *S. melongena* accessions of the Occidental group from the cluster formed by the Oriental group accessions plus A0416. On the other hand, the second axis, accounting for 20.7% of the genetic variation, separated the Indian accession MM1597 from the Chinese accession ASI-S-1 and the unknown origin accession A0416, as well as DH_ECAVI and IVIA-371 from H15 and AN-S-26 accessions.

### Transposon Dynamics

In order to understand the dynamics of transposable elements (TEs) during domestication, the class and number of TEs were compared between the seven cultivated *S. melongena* accessions and the wild *S. incanum* (**Table 4**). The average TE copy number in the seven cultivated accessions (1,090,121) was similar than in *S. incanum* (1,093,637). The latter had higher number of TEs than DH_ECAVI, IVIA-371, A0416, MM1597, and AN-S-26 but lower than ASI-S-1 and H15. For most of the TE families, ASI-S-1 and H15 had higher number of TE copies than *S. incanum* (**Supplementary Data S9**). In contrast, for the TE class of long terminal repeat (LTR)/Caulimovirus and DNA_nMITE/Harbinger, the *S. incanum* accession MM577 displayed the highest copy number.

**Table 4:** Statistics of the transposable elements identified in seven *Solanum melongena* and one *S. incanum* accessions of this study.

	*S. incanum*	*S. melongena*						
	MM577	MM1597	DH_ECAVI	AN-S-26	H15	A0416	IVIA-371	ASI-S-1
*ClassI//LTR/Copia*	92,218	91,834	91,623	91,435	92,270	91,516	91,553	93,767
*ClassI//LTR/Gypsy*	616,669	612,909	611,133	610,609	617,995	610,212	611,544	626,742
*ClassI//LTR/ltr_Others*	202,655	201,381	200,822	200,509	202,938	200,442	200,512	206,285
*ClassI//LTR/Caulimovirus*	4,842	4,756	4,671	4,666	4,759	4,702	4,777	4,797
*ClassI//nLTR/LINE*	70,446	70,491	70,285	70,283	70,697	70,457	70,189	71,092
*ClassI//nLTR/SINE*	17,916	17,905	17,863	17,854	17,892	17,893	17,796	17,978
*ClassII//DNA_MITE/hAT*	21,085	21,067	21,025	21,063	21,128	21,029	20,897	21,184
*ClassII//DNA_MITE/Tc*	31,303	31,330	31,214	31,192	31,332	31,301	31,065	31,427
*ClassII//DNA_MITE/MuDR*	0	0	1	0	0	0	0	0
*ClassII//DNA_MITE/mite_Others*	21,282	21,191	21,187	21,135	21,211	21,209	21,058	21,311
*ClassII//DNA_nMITE/MuDR*	7,719	7,710	7,691	7,659	7,738	7,676	7,627	7,841
*ClassII//DNA_nMITE/EnSpm*	7,489	7,513	7,500	7,470	7,505	7,480	7,474	7,556
*ClassII//DNA_nMITE/Harbinger*	13	2	3	2	1	4	2	2
**Total**	1,093,637	1,088,089	1,085,018	1,083,877	1,095,466	1,083,921	1,084,494	1,109,982

The TE size proportion of the genome was compared among the eggplant accessions and other main crops. Eggplant had similar TE proportion (average of 60.4% for the eight accessions) as the two major Solanaceae crops, tomato (63.2%) (Tomato Genome Consortium, 2012) and potato (54.4%) (Potato Genome Sequencing Consortium, 2011), but lower than pepper (76.4%) (Kim et al., 2014) (**Supplementary Data S10**). By comparing with Poaceae crops, TE proportion in eggplant was lower than maize (84.2%) (Schnable et al., 2009), similar to *Sorghum bicolor* (62%) (Paterson et al., 2009), and higher than rice (39.5%) (Yu et al., 2002). Interestingly, nearly all TEs in eggplant belongs to LTR (97.3%), representing a superfamily in class I TE, which is consistent with the high LTR proportion in tomato, potato, and pepper, but much higher than the three Poaceae crops (rice, sorghum, and maize).

## Discussion

In this study, we performed the first whole-genome resequencing analysis of eggplant, in which seven phenotypically diverse eggplant materials from different geographic regions and a wild relative were included. This pioneering resequencing study has significant relevance for eggplant breeding. In this way, these eight accessions are the founders of an eight-way MAGIC population that is currently under development and which may make a significant impact in eggplant breeding (Cavanagh et al., 2008; Gramazio et al., 2018). Pascual et al. (2015) emphasized the importance of the information generated by the resequencing of the founder parents of a tomato MAGIC population in the mapping precision of quantitative trait loci, even if many tools and molecular markers were already available in this crop. In addition, in the eggplant genepool, only a few studies have generated a significant set of molecular markers (Barchi et al., 2011; Gramazio et al., 2016; Acquadro et al., 2017), although they are not sufficient for a deep molecular characterization of a multiparent population and for building the corresponding saturated genetic linkage map from the founders (Pascual et al., 2015). In addition, no arrays, chips, or other genotyping platforms, as those available in other Solanaceae crops like tomato (Sim et al., 2012a; Víquez-Zamora et al., 2013), potato (Felcher et al., 2012; Vos et al., 2015), or pepper (Hill et al., 2013; Hulse-Kemp et al., 2016), have been developed so far in eggplant for high-throughput genotyping. Thus, one of the main aims of this study was to develop a large genome variation data set of polymorphic markers among the accessions that can serve as an a source of markers, from a diverse germplasm, for the potential development of genotyping platforms, breeding purposes, and further eggplant genetic and genomic studies including the genotyping of our MAGIC population.

### Data Quality and Mapping Rates

For this study, we have mapped the over 1.2 billion paired-end high-quality reads of the eight accessions against the new high-quality eggplant reference genome “67/3” (Barchi et al., 2019a). The high percentage of mapping rates of the eight lines (over 85%) confirms the high-quality of the “67/3” reference genome, although in other model species, the rates of unmapped reads were lower, such as the 3–5% in tomato (Causse et al., 2013; Aflitos et al., 2014) or 10–15% in rice (Subbaiyan et al., 2012; Xu et al., 2012). This might be due to different factors like the lower degree of the sequence assembly progress, the different level of repetitive elements, the genetic divergence between the sequenced accessions and the reference one, or the higher level of polymorphism, among others (Xu et al., 2012; Causse et al., 2013). However, the overall mapping coverage was around 20×, higher than the most common mapping coverage (around 10×) in resequencing studies of just a few years ago (Guo et al., 2013; Zhou et al., 2015), and encompassed practically the whole length of the reference genome for all the accessions except for *S. incanum*, in which the genome coverage onto the reference genome was on average 95%, evenly distributed for all the chromosomes. The *S. incanum* difference compared with the common eggplant accessions might be due to the phylogenetic distance between the two taxa, which is estimated in 1.56 MYA (Särkinen et al., 2013; Vorontsova et al., 2013; Acquadro et al., 2017) or to genome structural elements that are absent in *S. melongena* like repetitive sequences specific of *S. incanum* that difficult a sensitive mapping (Xu et al., 2012; Aflitos et al., 2014). This 5% of uncovered *S. incanum* genome may host important genic regions that have been lost during the domestication process, such as tolerance or resistance to stresses, as proposed by Aflitos et al. (2014) for tomato wild relatives. These authors also emphasized the need to focus efforts also in sequencing and assembly multiple reference genomes from crop wild relatives (CWRs) to avoid biased data interpretation and identify genome regions affected by genetic erosion. However, since the dynamics of gene gains and losses during domestication is not yet fully understood, other reasons may explain the lower genome coverage of *S. incanum*. In fact, among others, the 5% of uncovered regions could be also resulting from: i) regions gained by *S. melongena* during domestication by self-duplication, diversification or introgressions from other species, ii) regions present in the common ancestor of *S. incanum* and *S. melongena* that have been lost in *S. incanum*, and/or iii) hypervariable regions in *S. incanum* in which mapping using Burrows–Wheeler indexing may not be sensitive enough.

### Whole Genome Sequence Diversity

Our study has identified the largest set of polymorphisms to date in eggplant, consisting of almost 11 million of high-quality variants. Over 85% of these polymorphisms were SNPs, which are cheaper and easy to automate for high-throughput genotyping respect to other markers (Kim et al., 2016). In this respect, Yan et al. (2010) estimated that SNPs are on average 75% cheaper and 100-fold faster than simple sequence repeat gel-based methods to build a genetic map using a high-throughput genotyping platform. The whole-genome resequencing approach has been revealed as an excellent strategy to develop a large number of markers in many crops and mainly in their CWRs, where a general lack of information slows down their use in breeding programs and genetic studies (Aflitos et al., 2014; Brozynska et al., 2016). For example, 6 and 9 million SNPs were identified from wild and cultivated soybean accessions (Lam et al., 2010; Zhou et al., 2015), 6.5 million from wild rice accessions (Xu et al., 2012), and over 10 million from wild tomatoes (Aflitos et al., 2014; Gao et al., 2019). In these studies, the CWRs yielded up to 20-fold more variations compared to the heirloom and cultivated accessions, this without taking into account the genetic regions of CWRs that do not map onto the cultivated reference genome. Although in our study we just resequenced one wild relative (*S. incanum*), the results of the aforementioned studies are similar to ours, as *S. incanum* displayed a ninefold higher number of variations compared to cultivated common eggplants. The resequencing of more eggplant relatives will probably result in a greater difference in number of variants between the cultivated species and CWRs, particularly taking into account that many vegetables, including eggplant, have gone through severe genetic bottlenecks during domestication (Meyer et al., 2012; Meyer et al., 2015; Qi et al., 2013). Our study confirms that, as in other crops, the use of CWRs for introgression breeding can efficiently contribute to broadening the genetic basis of crops (Prohens et al., 2017). In this way, for several economically important crops like rice, tomato, or wheat, introgression breeding started several decades ago, and currently, many new varieties carry introgressed regions from CWRs (Dempewolf et al., 2017). For eggplant, the first efforts to harness the valuable large pool of genetic diversity of its wild relatives started just a few years ago thanks to the development of *ad hoc* breeding tools and strategies (Toppino et al., 2008; Liu et al., 2015; Kouassi et al., 2016; Plazas et al., 2016; Acquadro et al., 2017). The information produced in this study and the SNPs identified from *S. incanum* will accelerate the introgression of genomic regions of this drought-tolerant species (Knapp et al., 2013; Gramazio et al., 2017a) to develop new resilient eggplant varieties.

In addition, in our study, we have identified a large number of high-confidence sets of polymorphisms in common eggplant accessions, providing valuable information for performing high-throughput genotyping in cultivated or wild germplasm through the development of arrays, chips, or other high-throughput genotyping platforms. In this way, a first genotyping platform in eggplant, based on the single primer enrichment technology (SPET), was recently developed with the information produced in this study (Barchi et al., 2019b). The eggplant SPET platform, which consists of 5,093 probes designed from the SNPs identified throughout the genome of the eight eggplant accessions resequenced in our study, has been already used to genotype the diversity of a germplasm collection of 422 cultivated eggplant and CWR accessions (Barchi et al., 2019b). The results from the screening of a large eggplant germplasm dataset including eggplant CWRs using the polymorphisms identified in this study demonstrated their transferability in the assessment of closely related species from different genepools with high interest in breeding, as well as their potential a wide variety of studies like phylogenetic, domestication, or genome-wide association studies (Barchi et al., 2019b).

### Relationships Among the MAGIC Population Founders and Candidate Introgression Detection

In this first eggplant resequencing study, we tried to maximize the phenotypic diversity for important breeding traits coupled with genetic diversity and the geographic origins. The genetic distance matrix and the PCoA obtained with the entire SNP dataset confirmed our selection criteria. In fact, the higher genetic identity values were between H15 and AN-S-26, which share some common traits like long calyx and being used for making pickles (Muñoz-Falcón et al., 2009a), and between IVIA-371 and DH_ECAVI, both having an élite background and with large fruit (Hurtado et al., 2014; Gramazio et al., 2017b). These four accessions clustered together since they were chosen to represent the Occidental diversity of eggplant (Vilanova et al., 2012). However, despite the phenotypic diversity, relatively low genetic diversity has been found among them, suggesting that the European accessions might have been diversified from a reduced genepool (Muñoz-Falcón et al., 2008; Muñoz-Falcón et al., 2009a; Muñoz-Falcón et al., 2009b; Meyer et al., 2012; Cericola et al., 2013). ASI-S-1 accession represented the Chinese domestication center and displays a great phenotypic similarity with the eggplant reference genome “67/3,” showing rounded and purple fruits and the recessive allele for the dominant pigment under calyx trait (Toppino et al., 2016). This similarity is confirmed by the lowest number of polymorphisms, compared to the rest of the accessions. Regarding accession A0416, the selection was made exclusively based on its peculiar phenotype, flattened ribbed white fruits, since its origin was unknown. Our results suggest that this accession might belong to the Oriental eggplant genepool (Vilanova et al., 2012) and, based on PCoA results, probably closer to the Chinese domestication center than to the Indian center (Meyer et al., 2012). Finally, the accession MM1597, representing the Indian domestication center, is closer to the European group than the Chinese accession. In this way, it has been suggested that Arab traders brought eggplants from southeast Asia into Europe in the 14th century (Lewicki et al., 1974; Daunay and Janick, 2007; Meyer et al., 2012).

An alternative way to visualize the relationships among the accessions could be done by analyzing the distribution of variants (Aflitos et al., 2014), which was uneven along the whole genome. More than 90% of the polymorphisms were found in intergenic and noncoding intragenic regions, being the variants in coding regions <9% on average. Similar ratios have been also observed in other crops (Xu et al., 2012; Causse et al., 2013; Aflitos et al., 2014; Zhou et al, 2015). Large differences were found among the 12 chromosomes in the number of polymorphisms, although the number of polymorphisms was highly related to the physical length of them. Differences were also observed among the accessions for a given chromosome. Interestingly, some accessions shared genomic regions with a similar large density of SNPs, which visually are like large blocks of peaks when the SNPs are divided and plotted into 10-Mbp sized bins. This similar SNPs distributions among some accessions have been interpreted as candidate introgressions and could help in reconstructing the breeding history of a crop (Sim et al., 2012b; Causse et al., 2013; Aflitos et al., 2014). In this study, footprints of old intraspecific and, more likely, interspecific hybridizations were identified in five chromosomes (2, 6, 9, 11, and 12) and more clearly in homozygous than in the heterozygous variants. The patterns of shared blocks of peaks in the Occidental accessions suggests that they share common ancestors, with many common candidate introgressions not only between IVIA-371 and DH_ECAVI, AN-S-26 and H15, but also among H15, IVIA-371, and AN-S-26. Furthermore, this alternative approach provides additional evidence to support that A0416 could have originated in the Chinese domestication center because of the general similarity in the pattern of SNPs databases and the similar distribution of SNPs in the first 70 Mbp of chromosome 6.

The analysis of the Gene Ontologies associated with genes with “high” impact variants, which were found in either homozygous and heterozygous state, shows a common trend in all the accessions related with variation on the photosynthetic pathway. Most of these genes, such as “ribulose bisphosphate carboxylase large chain” (e.g., SMEL_002g155400.1, SMEL_005g233370.1, and SMEL_012g386960.1) should be located in the chloroplast genome, but in these cases, there are located in the nuclear genome, in specific chromosomal locations. Chloroplast genes are commonly transferred to the nuclear genome by different mechanisms producing nuclear integrants of plastid DNAs (Michalovova et al., 2013). These chloroplast genomic fragments are under strong purifying selection, so it is not surprising that these genes present “high” impact variants that drive to a fast removal of the possible activity these genes. More intriguing is the high percentage of genes with “high” impact variants associated with the DNA maintenance and restoring after damage (mostly helicase-like proteins) machinery that can be found in *S. incanum* like “telomere maintenance” (GO:0000723, 25 out of 58 genes) and “DNA repair” (GO:0006281, 41 out of 195 genes). In plants, helicase-like proteins play a critical role in stress tolerance responses (Nidumukkala et al., 2019). Natural mutations and overexpression of DEAD box helicases confirmed the role of this subgroup of helicases in providing stress tolerance, like cold or salinity (Gong et al., 2002; Vashisht et al., 2005; Shivakumara et al., 2017; Mahesh and Udayakumar, 2018). As well, many RNA modification genes (mostly RNA methyltransferases and ribonucleases), related to transcriptional and posttranscriptional activities, presented “high” impact effect variants in *S. incanum* like “transcription by RNA polymerase III” (GO:0006383, five out of eight genes), “mRNA cleavage” (GO:0006379, two out of two genes), “rRNA modification” (GO:0000154, three out of five genes), “ncRNA processing” (GO:0034470, 20 out of 80 genes), “RNA methylation” (GO:0001510, 5 out of 15 genes), and “RNA 3’-end processing” (GO:0031123, 6 out of 18 genes). RNA methyltransferases and ribonucleases, along with many other epigenetic activities, and protein kinase (GO:0043549, 4 out of 17 genes) are reported to be involved in plant abiotic stress response (Deng et al., 2013; Wang et al., 2017, Hu et al., 2019). In addition, three out of four genes that encode for guanosine diphosphate mannose, which is a precursor for ascorbate, presented “high” impact variants. Ascorbic acid has important antioxidant and metabolic functions among which is environmental stress adaptation (Wheeler et al., 1998; Bao et al., 2016). The *S. incanum* accession MM577 was collected in a desertic area in Israel, where day and night temperatures differ considerably and plants experimented heat and cold stress, along with extreme drought conditions. It has already been reported that *S. incanum* is highly tolerant to drought and to some fungal diseases (Knapp et al., 2013), but no studies have been performed so far on its tolerance to other abiotic stresses. Based on these results, investigating the response of this wild species to other stresses, like heat, cold, and salinity, in order to introduce it in breeding pipelines, the advanced backcross materials with elite genetic background already developed using *S. incanum* as wild donor (Gramazio et al., 2017a).

Regarding the other *S. melongena* accessions, “high” impact effect variants were found in MM1597 for “response to nitrate” (GO:0010167 and GO:0015706, two out of two genes) affecting the gene SMEL_003g189360.1 “high-affinity nitrate transporter 3.1.” Nitrogen is one of the major limiting factors for plant growth and crop yield, and in the past decades, nitrogen fertilization has increase more than 20-fold with high economic and ecological costs (Marschner, 2011). At present, the improvement of nitrogen use efficiency is an agriculture challenge to maintain high crop yield under low nitrogen for more sustainable agriculture. Measuring the nitrogen uptake of this accession in order to associate these variants with a different absorption capacity may provide information relevant for improving nitrogen use efficiency. In DH_ECAVI, two out of four genes presented “high” impact variants in “TOR signaling” (GO:0031929), an evolutionarily conserved protein kinase that is involved in the growth processes of cotyledons, true leaves, petioles, and primary and secondary roots (Shi et al., 2018). In H15, a “high” impact effect variant in the Myb transcription factor “leaf formation” (GO:0010338) could be associated with the smaller leaves and fruit pedicel compared to its related Andalusian eggplants (Muñoz-Falcón et al., 2009a). Accession A0416 had 7 out of 54 genes related to “recognition of pollen” (GO:0048544) that could partially explain the difficulty of obtaining hybrids when it is used as a female parent. However, all the “high” impact effect variants of **Supplementary Data 6** and **7** may be further investigated using our MAGIC population, which currently is under development, where the segregating recombinant lines will provide a more powerful resolution to dissect the genetics of many traits and associated variants with specific phenotypes.

### Transposable Elements

TEs make up the vast majority of all investigated plant DNA, and TEs’ dynamic changes (activation or purification) in a genome frequently influence their insertions, duplications, and the genome size (Levin and Moran, 2011). This dynamic TE is commonly influenced during domestication. When comparing the copy number of *S. melongena* accessions with the wild accession of *S. incanum*, two of them had higher TE copy and the other five had lower TE copy number than the *S. incanum* accession, suggesting possible dynamic TE events occurring in the course of domestication. Two families including LTR/Caulimovirus and DNA_nMITE/Harbinger in the wild accession were identified to be purified potentially underlying selection influences. In rice, a miniature inverted-repeat TE (MITE/mPing) amplified from ∼50 to ∼1,000 copies in four rice strains, and 70% of the 280 inserted TEs were within 5 kb of the coding regions (Naito et al., 2006). Two MITE families in *Medicago truncatula* produced highly polymorphic insertion sites in 26 ecotypes. In this latter study, a subset of insertions was present only in a cultivar, compared to the other 25 wide ecotypes, indicating activations of these two families during *M. truncatula* domestication (Grzebelus et al., 2009). Some TE insertions underlying the dynamics altered plant phenotype simply by inducing the preexisting expression of genes selected during domestication. Overexpression of *teosinte branched1* (*tb1*) in maize can induce a dramatic reduction in branch number relative to the progenitor species, resulting from a retrotransposon inserted in the promoter region of tb1 (Studer et al., 2011). These observations in eggplant suggest possible important roles of TEs during its domestication. However, in order to gain greater accuracy and sensitivity, we suggest resequencing further accessions of eggplant CWRs so as to identify TE copy number variations with statistical significance between the wild and cultivar accessions.

## Conclusions

The progressive availability of high-quality reference genomes and the continuous improvements of high-throughput sequencing technologies are fostering resequencing studies even for no-model plant species, including eggplant. In this study, we performed the first resequencing of a set of eggplant accessions, identifying the largest set of polymorphisms so far in eggplant genepool using accessions phenotypically and genetically very diverse. The usefulness and transferability of these polymorphisms identified has already been demonstrated by developing the first high-throughput genotyping SPET platform in eggplant, which has been used to assess the genetic relationships among domesticated accessions and wild related from several species in a large eggplant germplasm dataset. All the variants were structurally and functionally annotated and their effects predicted, which will be very useful for a wide type of studies and approaches like genomic editing or the characterization of natural, segregating (like the MAGIC population that we are currently developing with the accessions resequenced in this study), and mutants populations. In addition, the distribution of the variants has revealed footprints of putative ancient introgressions that can help to shed light on the yet unclear eggplant domestication history. The analysis of the transposon composition across the different accessions reveals different trends among eggplant accessions with some of them having more and others less TE than the wild relative *S. incanum*. Further experiments increasing the number of accessions should be done in order to elucidate the impact of the TE in the eggplant domestication process.

This first resequencing study in eggplant has gathered relevant genomic data and information that will be extremely useful for the development of a new generation of improved eggplant varieties adapted to present and future challenges in eggplant production and with better fruit quality. The information obtained will also be highly relevant for answering a plethora of scientific and technical questions and needs, including allele and variants discovery, germplasm genomic characterization, marker-assisted mapping for dissecting agronomic-associated loci, or providing insight into underlying molecular mechanisms of eggplant genome evolution, among others. In this way, this first study paves the way and encourages other researchers and breeders to perform the resequencing of more eggplant accessions, especially CWRs, where many valuable alleles for eggplant breeding have still to be discovered and exploited.

## Data Availability Statement

The datasets generated for this study can be found in the NCBI Short Read Archive under submission identifier SUB2829594 with the Bioproject identifier PRJNA392603. Raw reads of each accession are deposited under the accession numbers from SRR5796636 to SRR5796643. VCF files with the corresponding variants identified are available upon request to the corresponding author

## Author Contributions

PG, SV, JP, and AB conceived and designed the research. PG, HY, and TH analyzed the data. PG wrote the manuscript. PG, HY, TH, SV, JP, and AB reviewed and edited the manuscript. All authors read and approved the manuscript.

## Funding

This work has been funded by the European Union’s Horizon 2020 Research and Innovation Programme under grant agreement no. 677379 (G2P-SOL project: Linking genetic resources, genomes, and phenotypes of Solanaceous crops), by the Spanish Ministerio de Economía, Industria y Competitividad and Fondo Europeo de Desarrollo Regional/European Regional Development Fund (grant AGL2015-64755-R), and by the Spanish Ministerio de Ciencia, Innovación y Universidades (MCIU), Agencia Estatal de Investigación (AEI), and Fondo Europeo de Desarrollo Regional/European Regional Development Fund (grant RTI2018-09592-B-100). PG is grateful to Universitat Politècnica de València and to Japan Society for the Promotion of Science for their respective postdoctoral grants [PAID-10-18 and FY2019 JSPS Postdoctoral Fellowship for Research in Japan (Standard)].

## Conflict of Interest

The authors declare that the research was conducted in the absence of any commercial or financial relationships that could be construed as a potential conflict of interest.
