# Supplementary material for: Whole-Genome Resequencing of Seven Eggplant (Solanum melongena) and One Wild Relative (S. incanum) Accessions Provides New Insights and Breeding Tools for Eggplant Enhancement
Source: Front Plant Sci. 2019 Oct 8;10:1220. doi: 10.3389/fpls.2019.01220 (PMC6791922; doi:10.3389/fpls.2019.01220)

Supplementary data S3. Distribution of homozygous (red) and heterozygous (blue) polymorphisms along all the chromosomes for the seven *S. melongena* and one *S. incanum* (MM577) accessions resequenced identified using the *S. melongena* accession "67/3" as a reference genome (Barchi et al., 2019a). The peaks represent high frequencies of polymorphisms in a window size of 10 Mbp. The dashed lines of the same colour indicate similar patterns of polymorphisms distribution.

Chromosome 1 homozygous variants

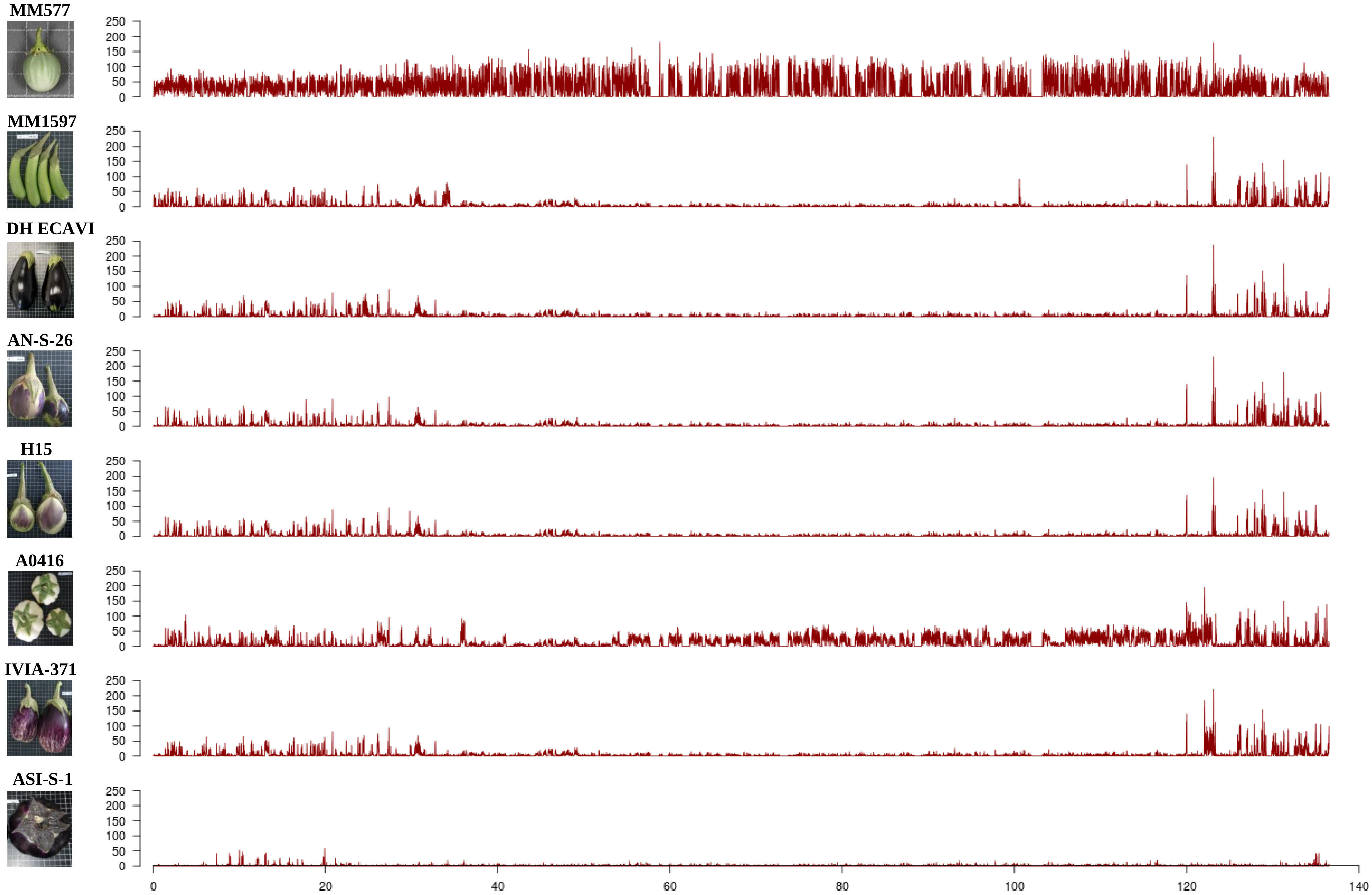

Chromosome 2 homozygous variants

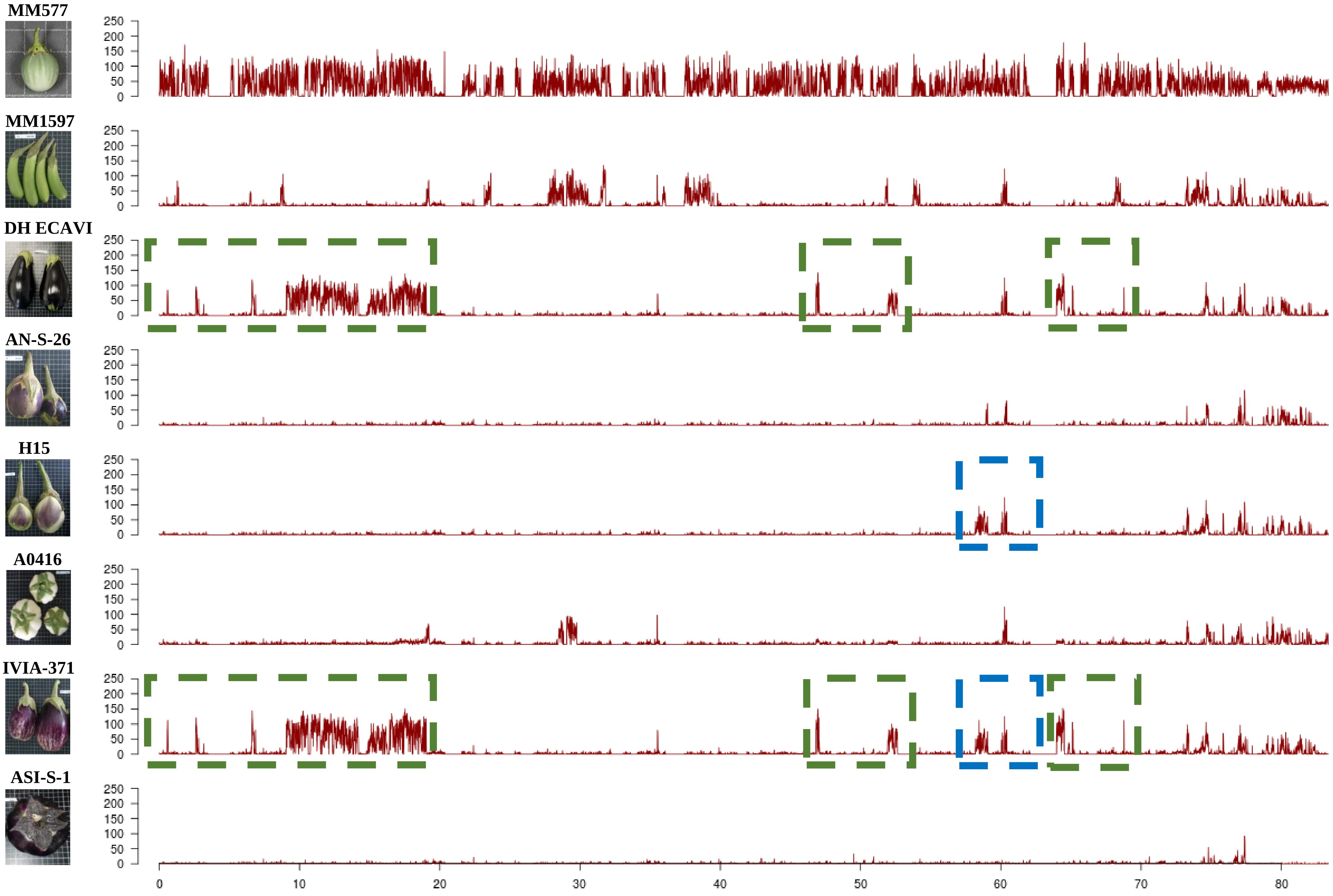

Chromosome 3 homozygous variants

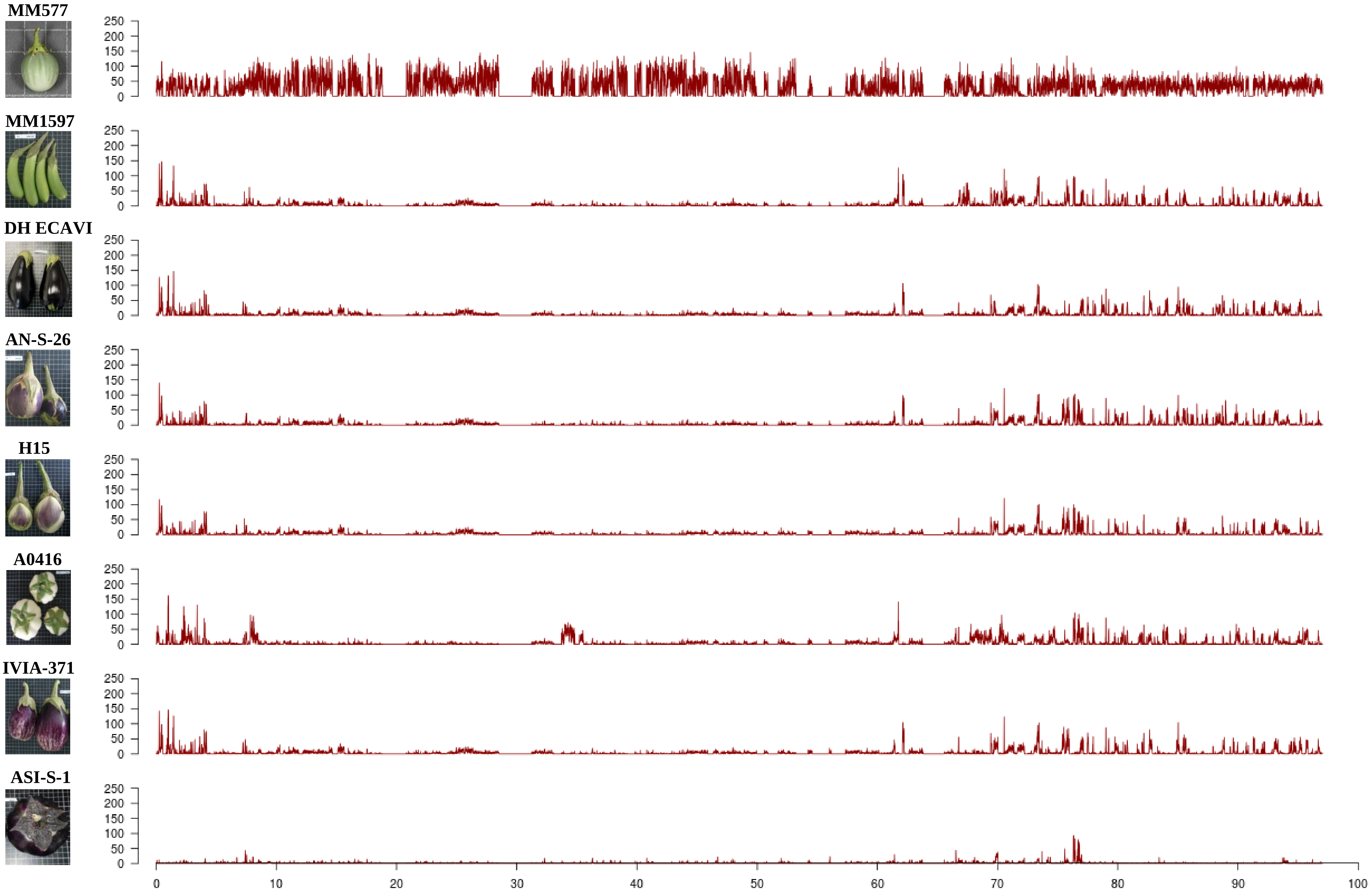

Chromosome 4 homozygous variants

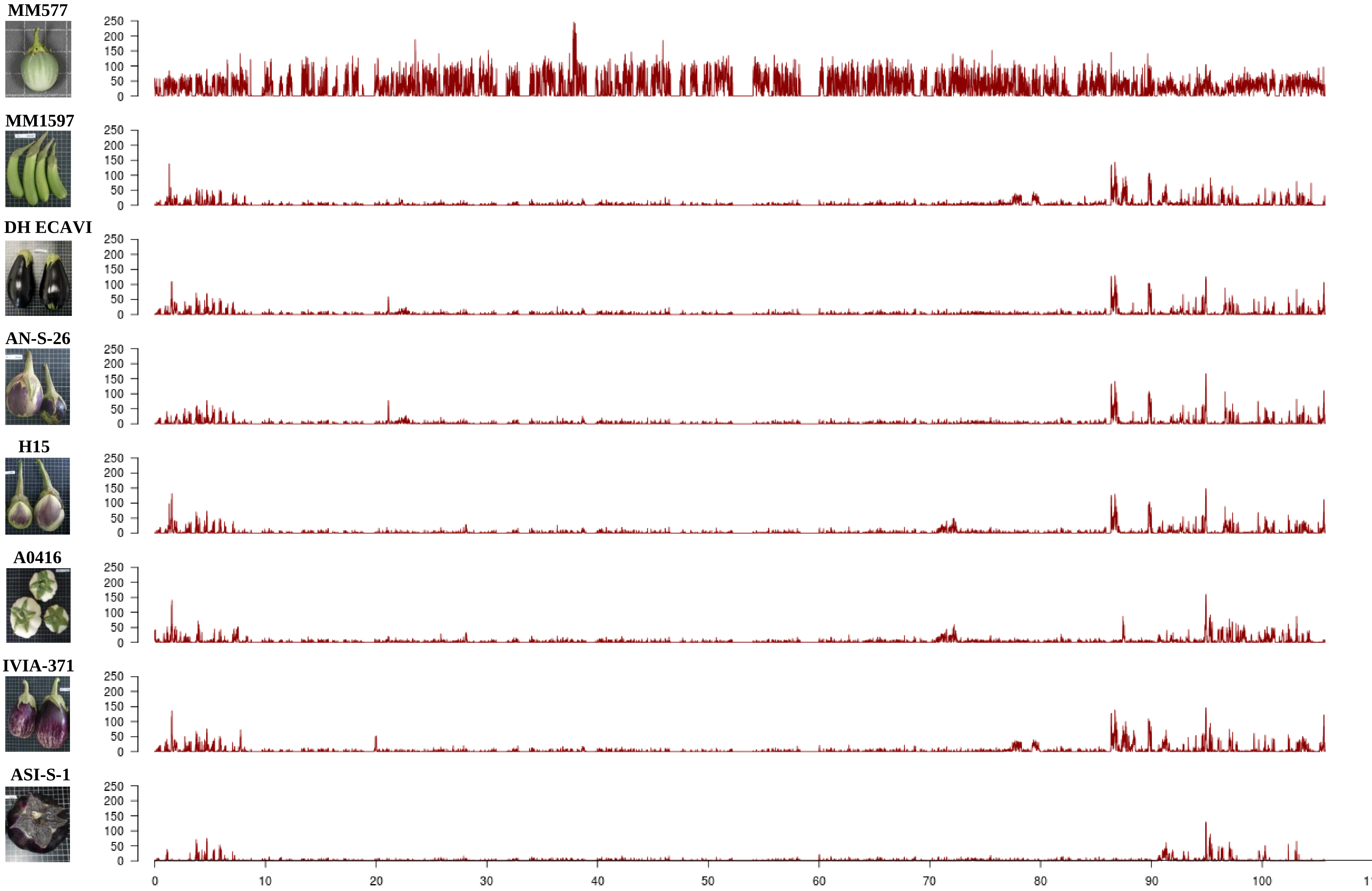

Chromosome 5 homozygous variants

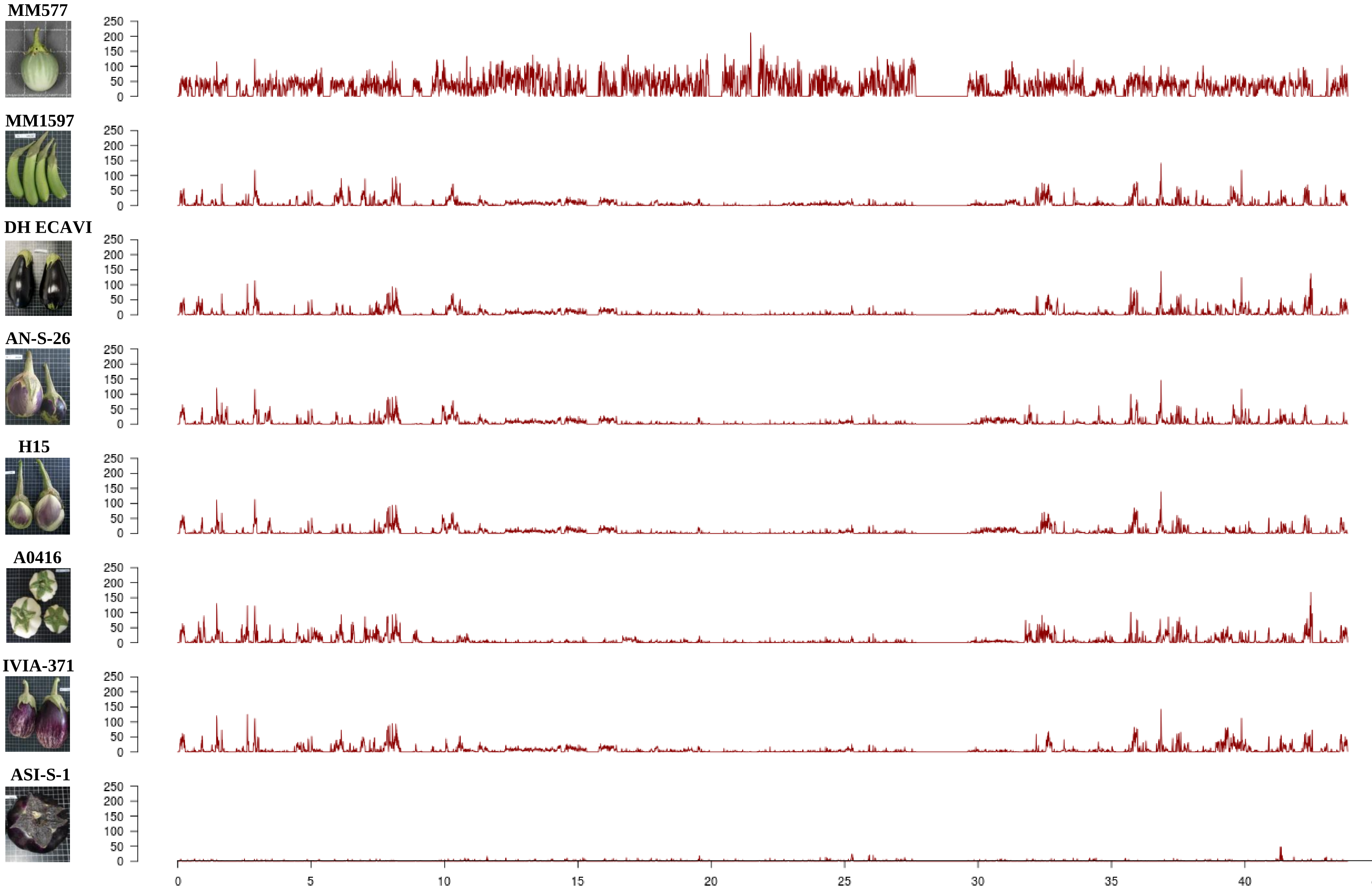

Chromosome 6 homozygous variants

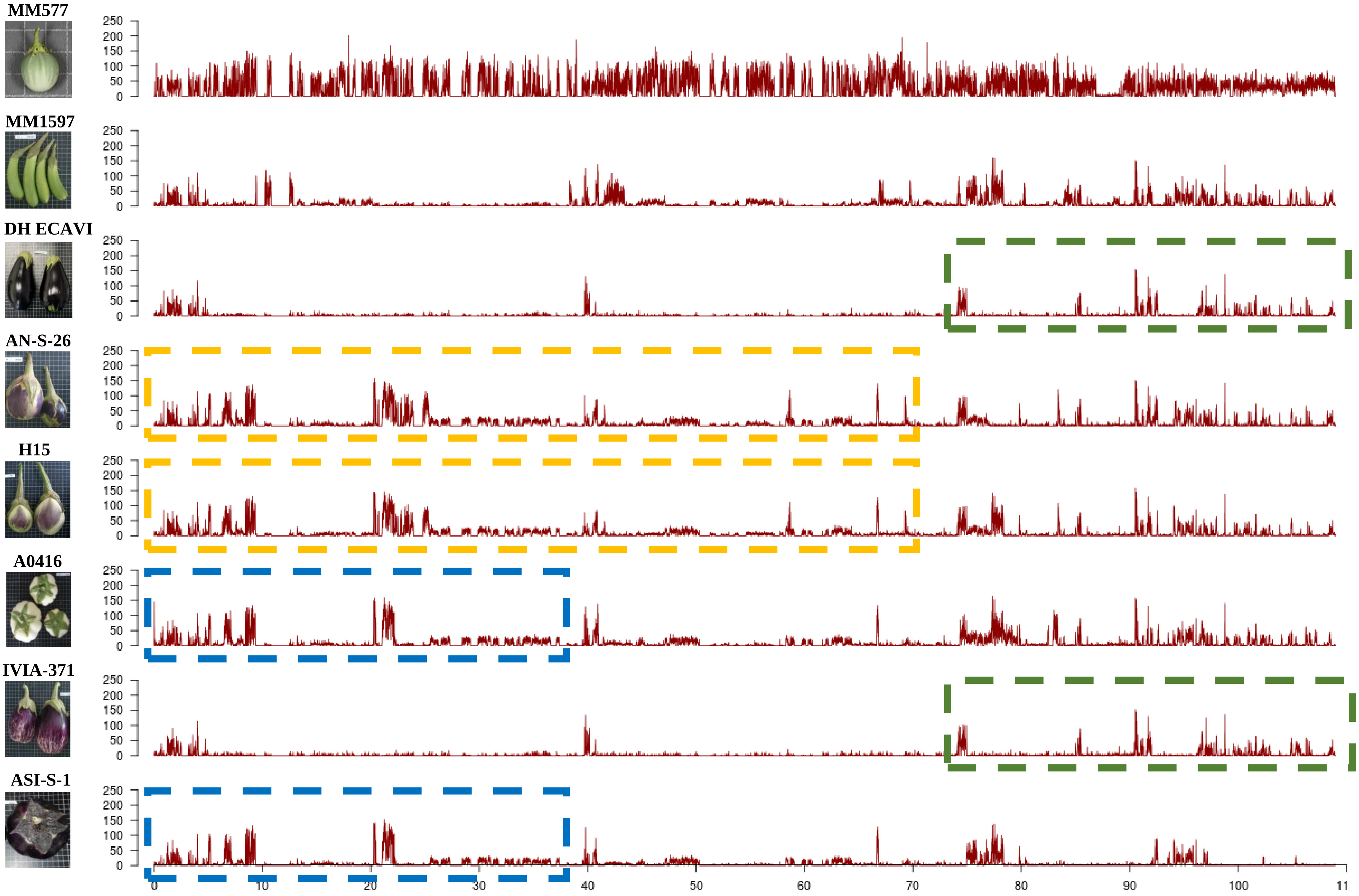

Chromosome 7 homozygous variants

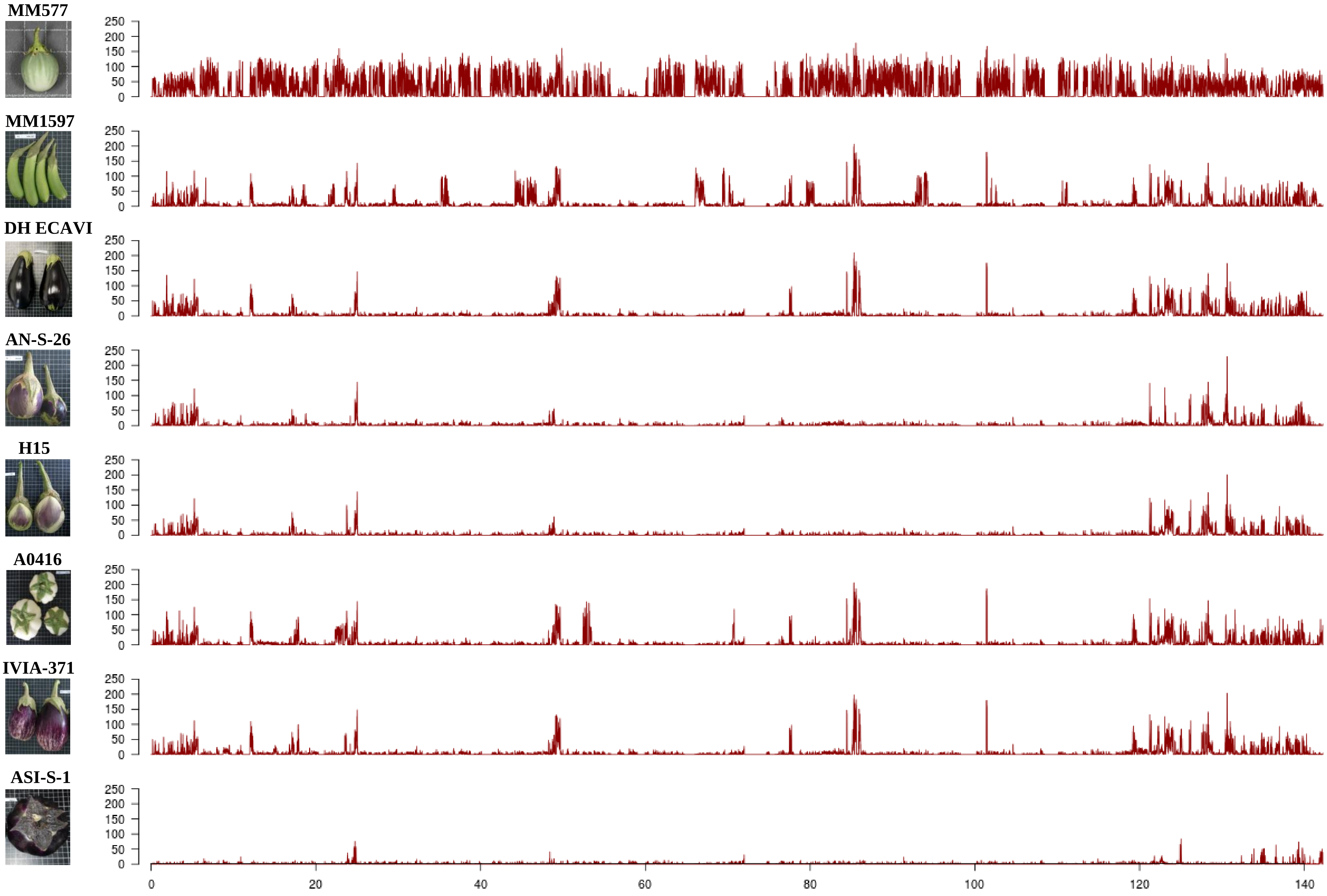

Chromosome 8 homozygous variants

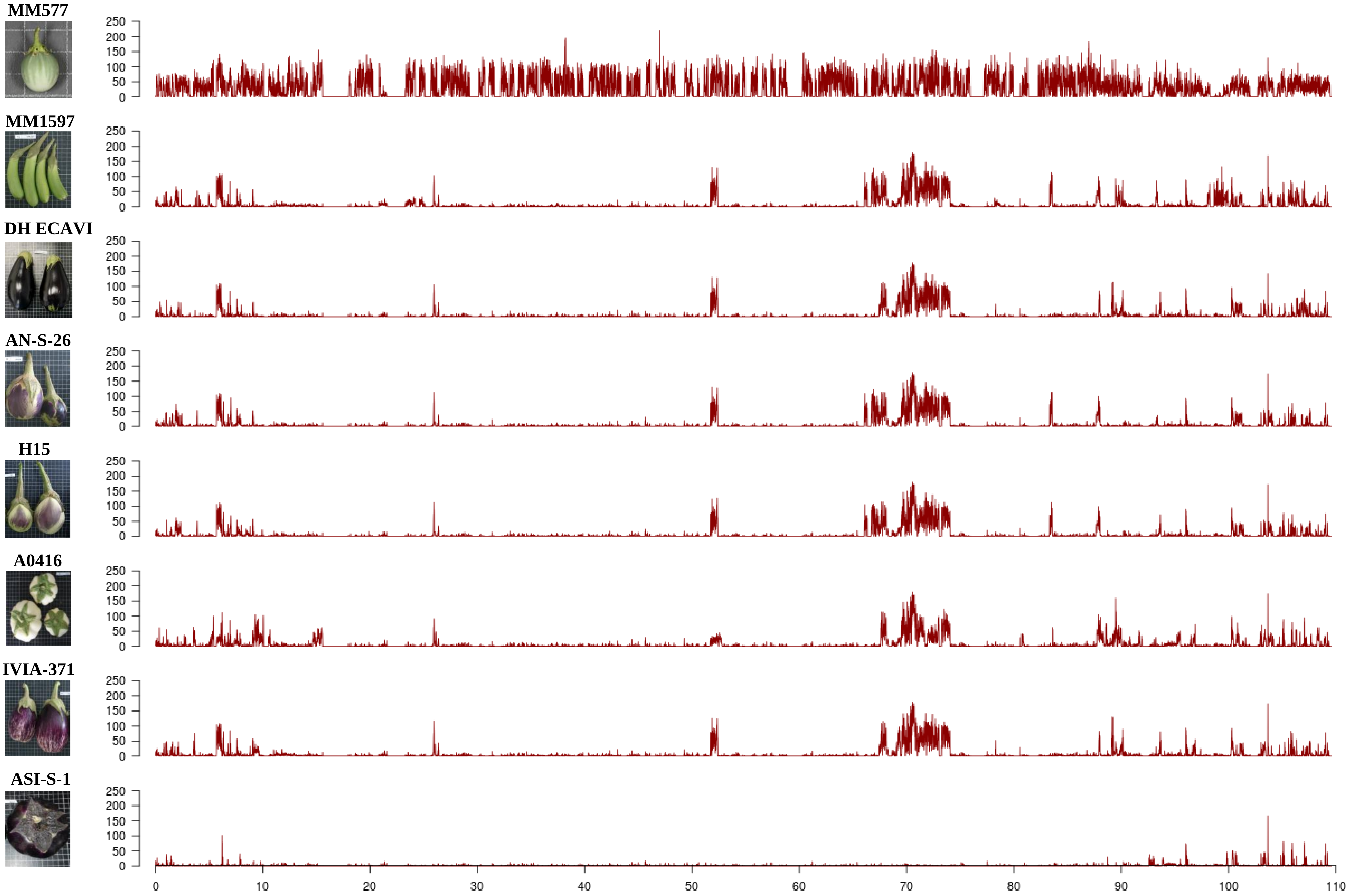

Chromosome 9 homozygous variants

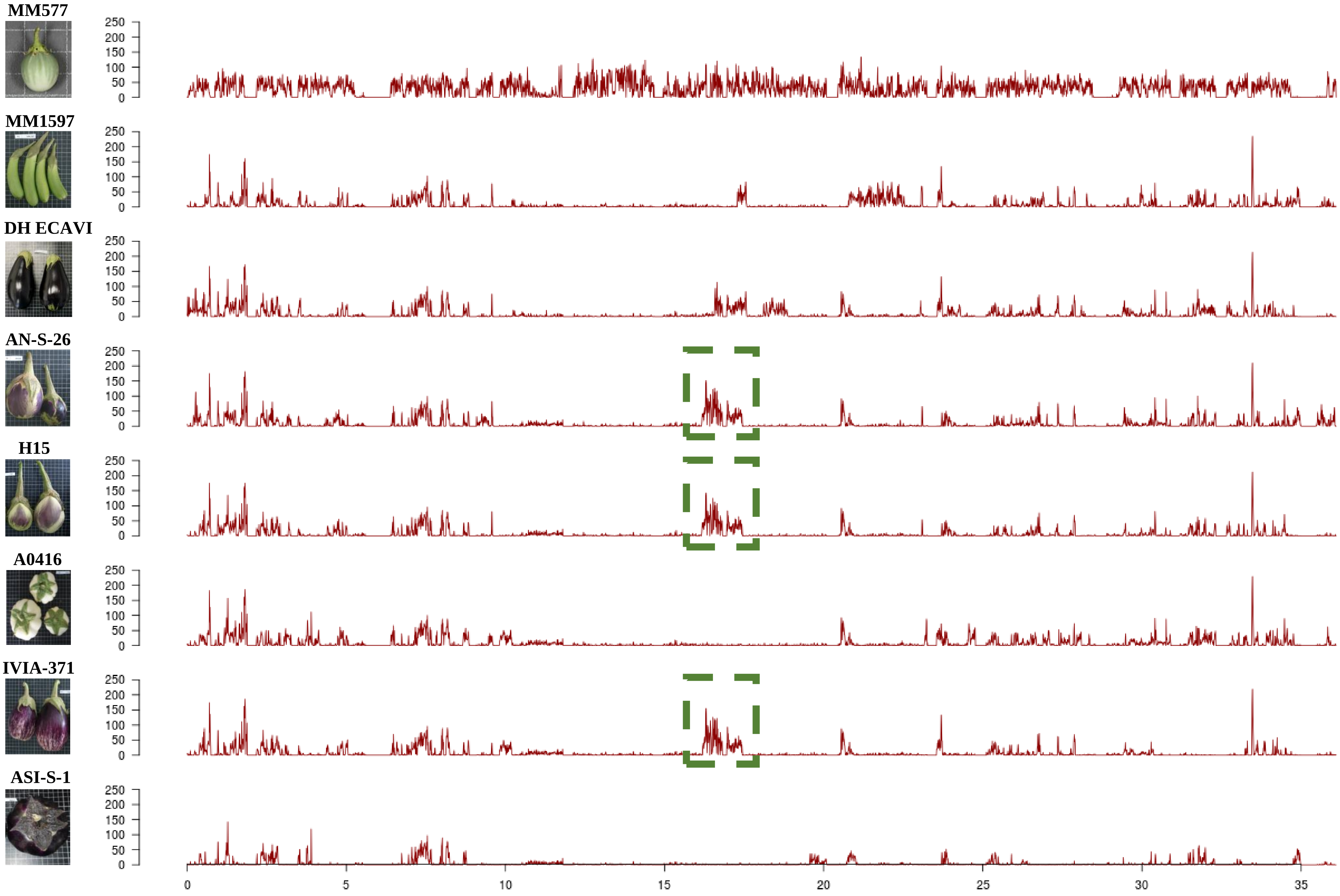

Chromosome 10 homozygous variants

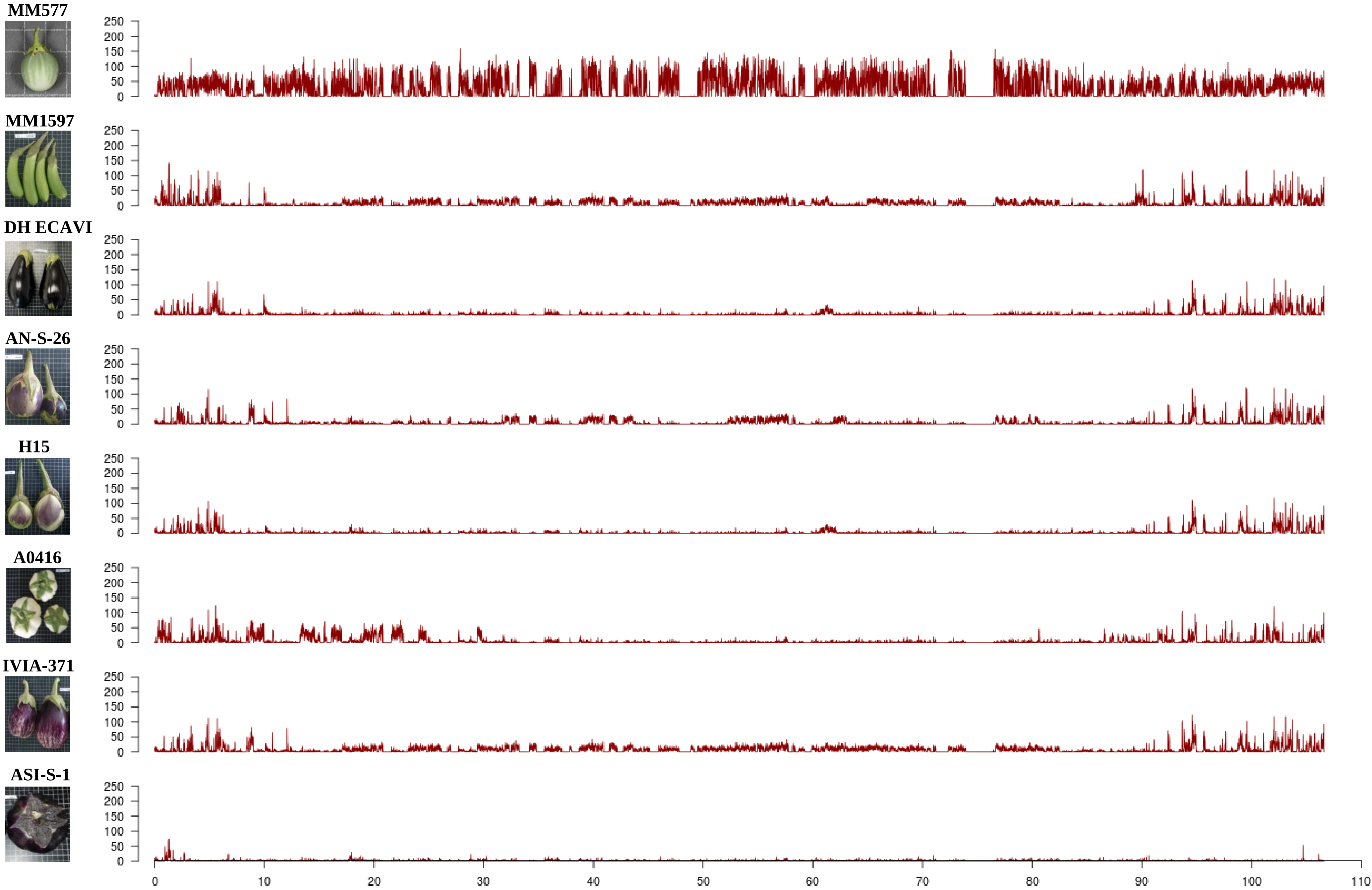

Chromosome 11 homozygous variants

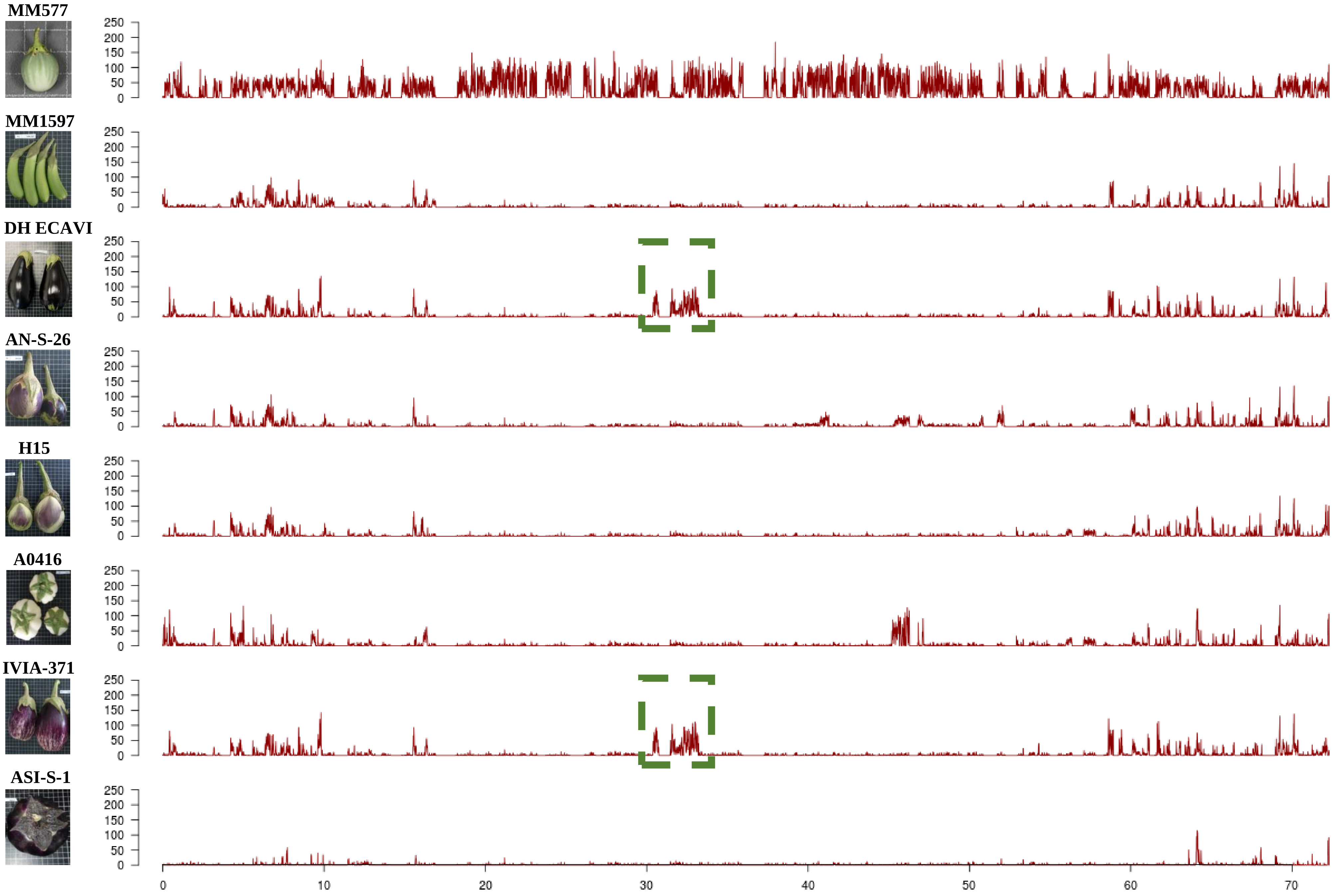

Chromosome 12 homozygous variants

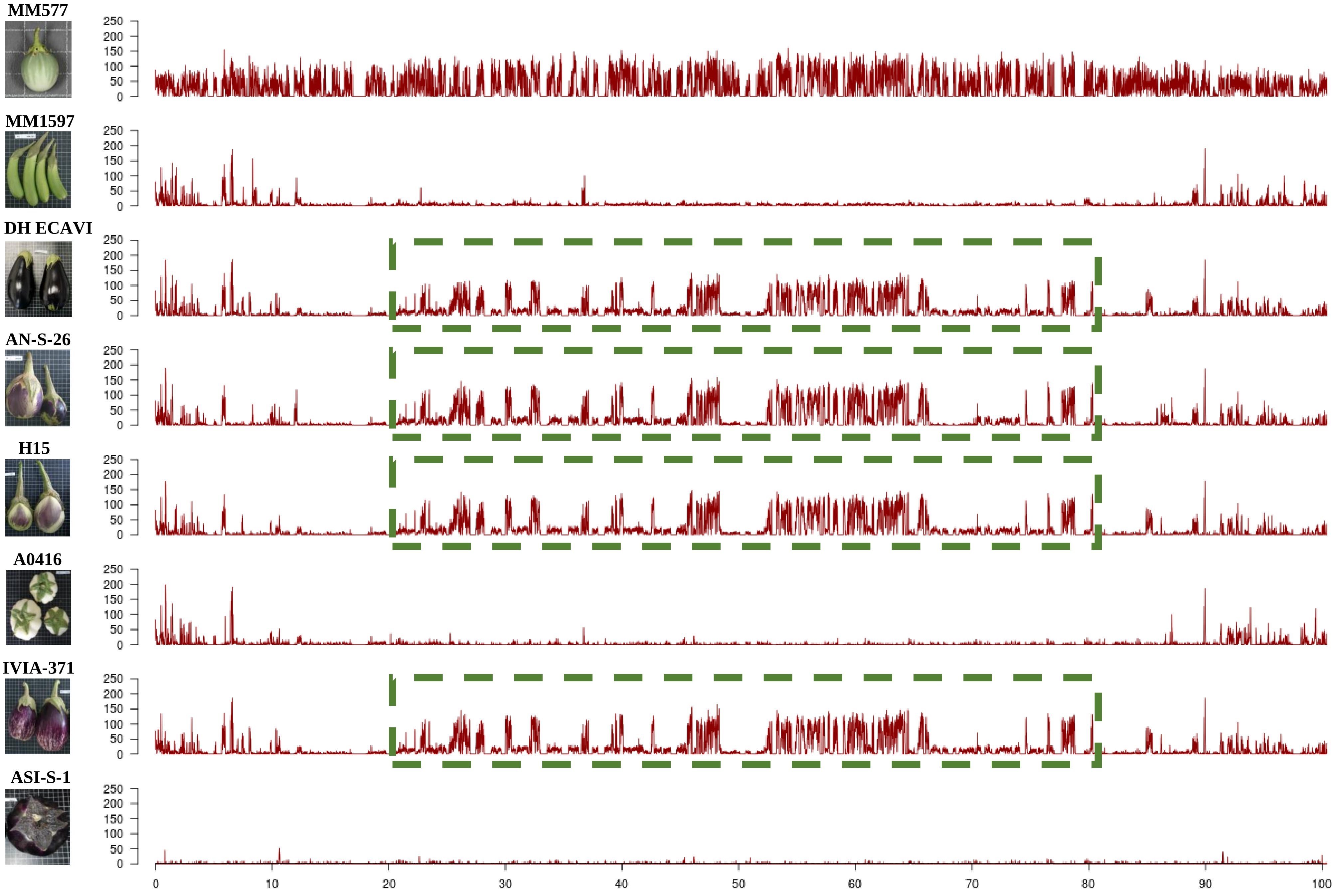

Chromosome 1 heterozygous variants

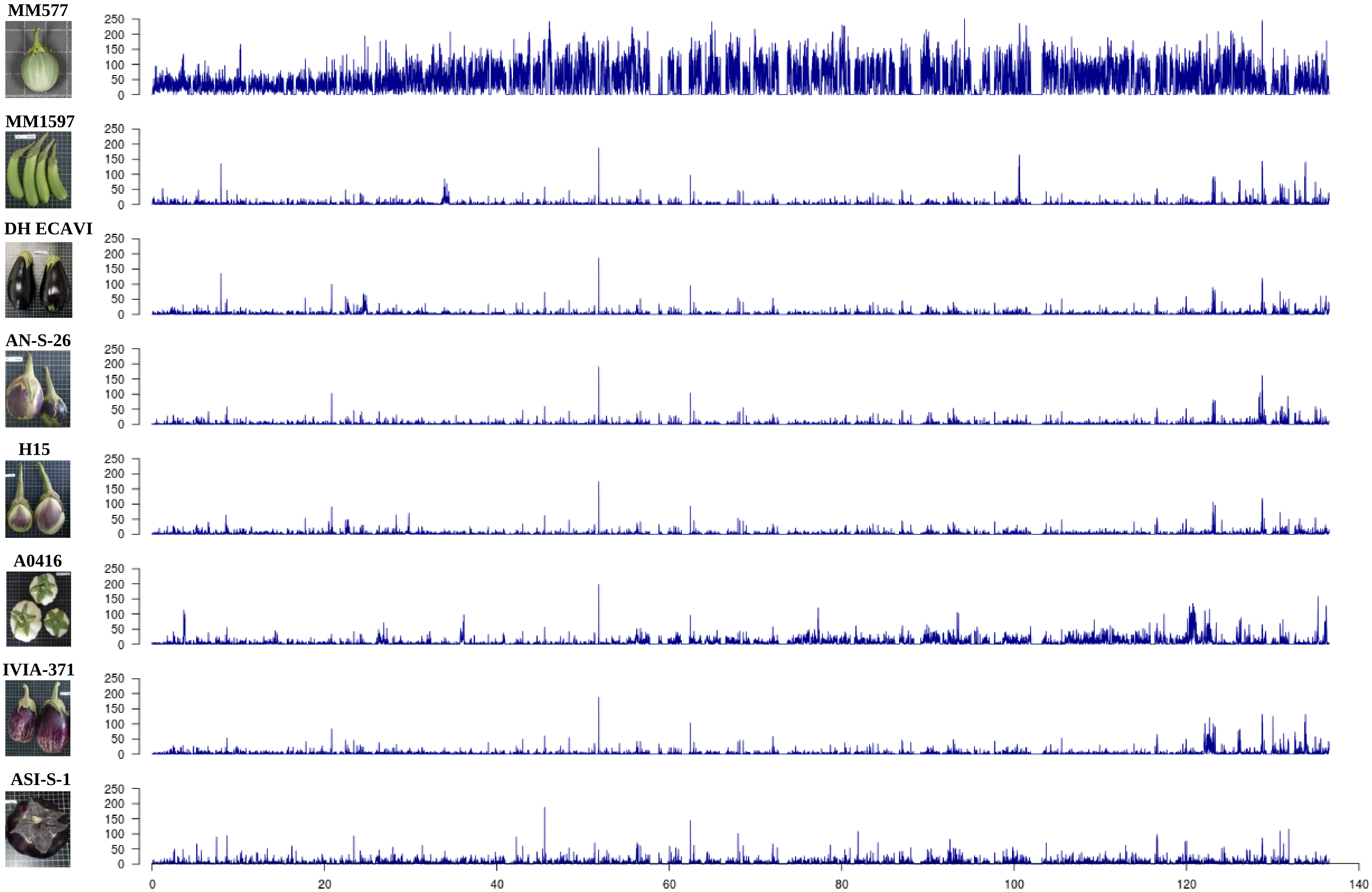

Chromosome 2 heterozygous variants

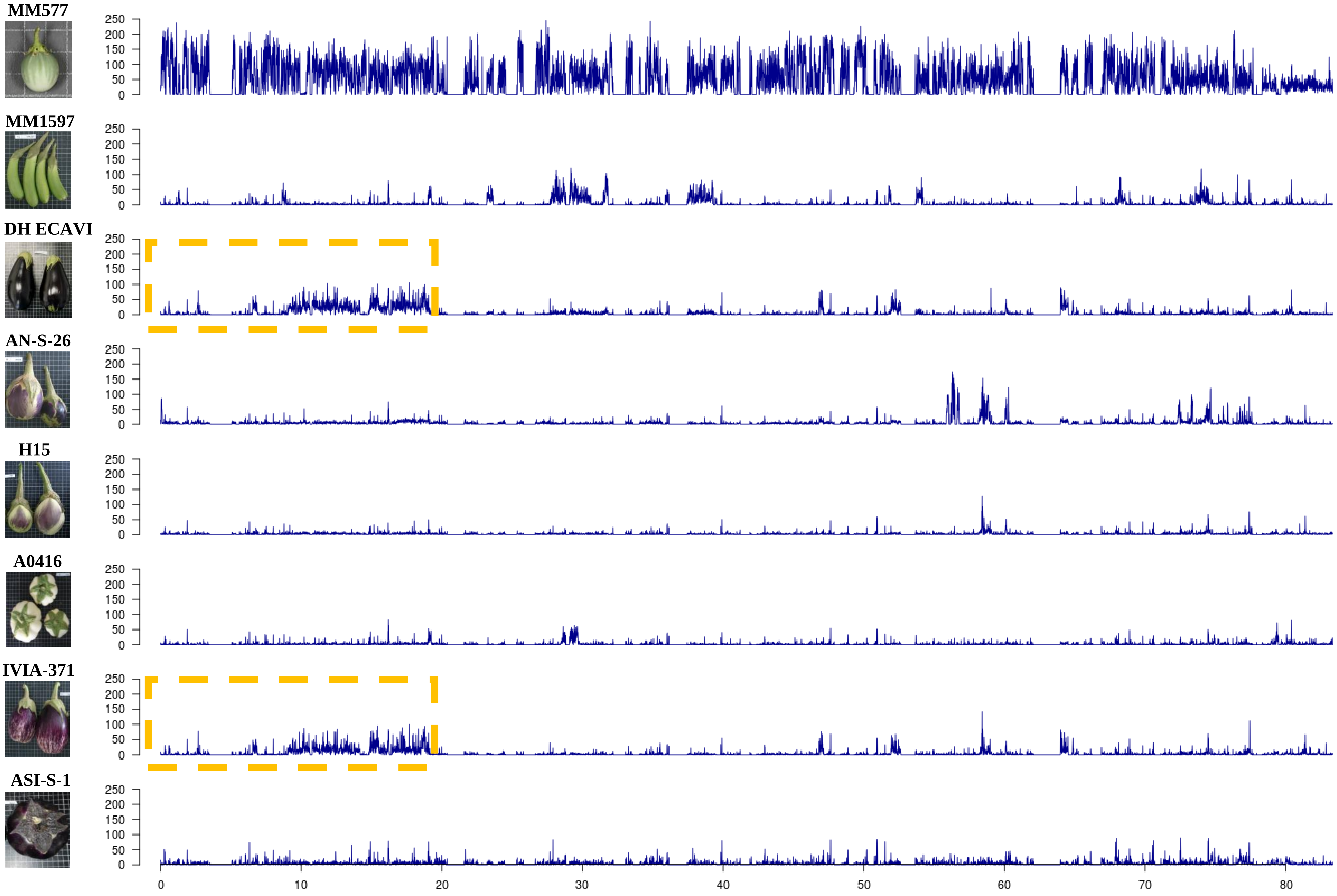

Chromosome 3 heterozygous variants

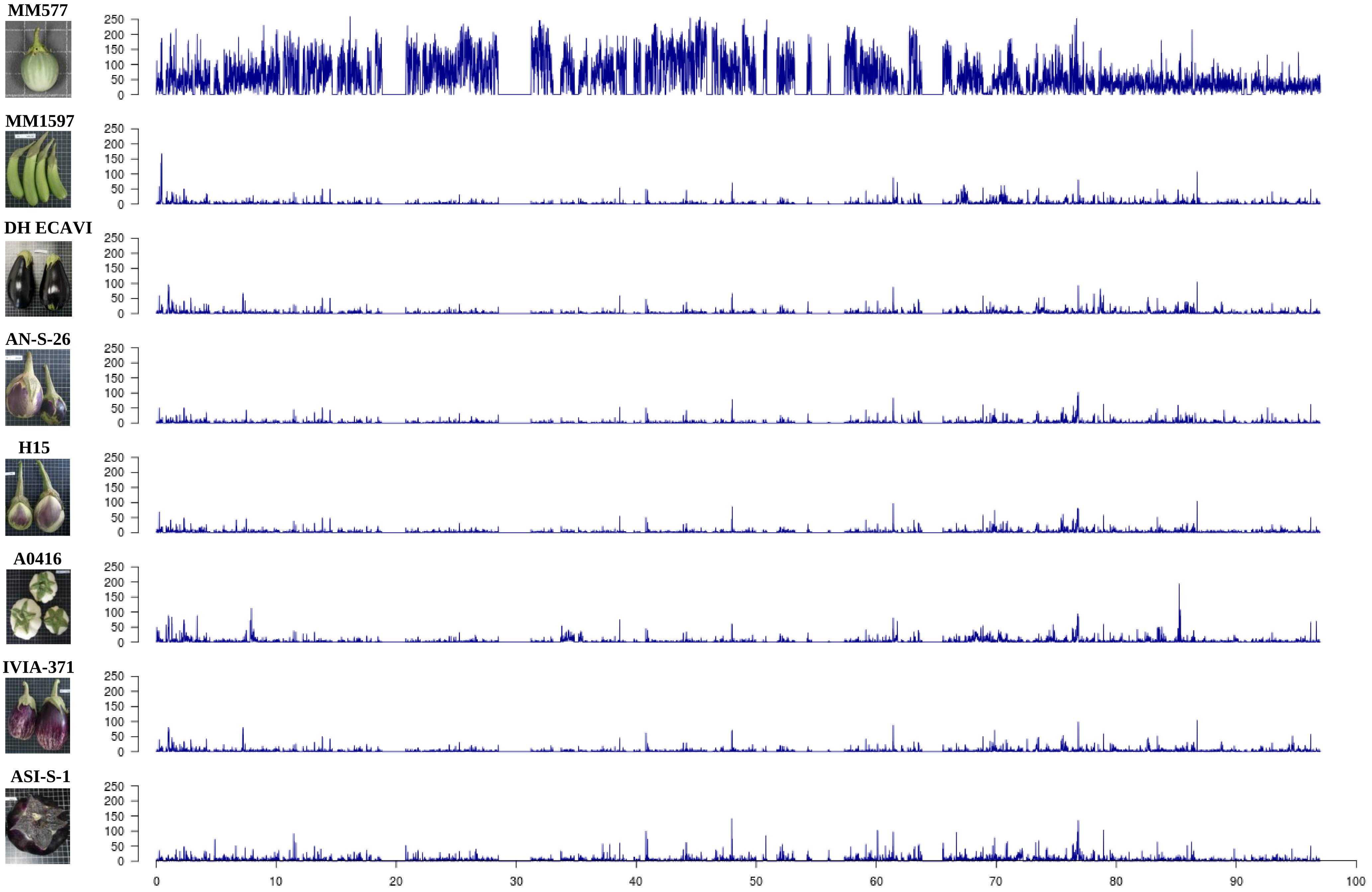

Chromosome 4 heterozygous variants

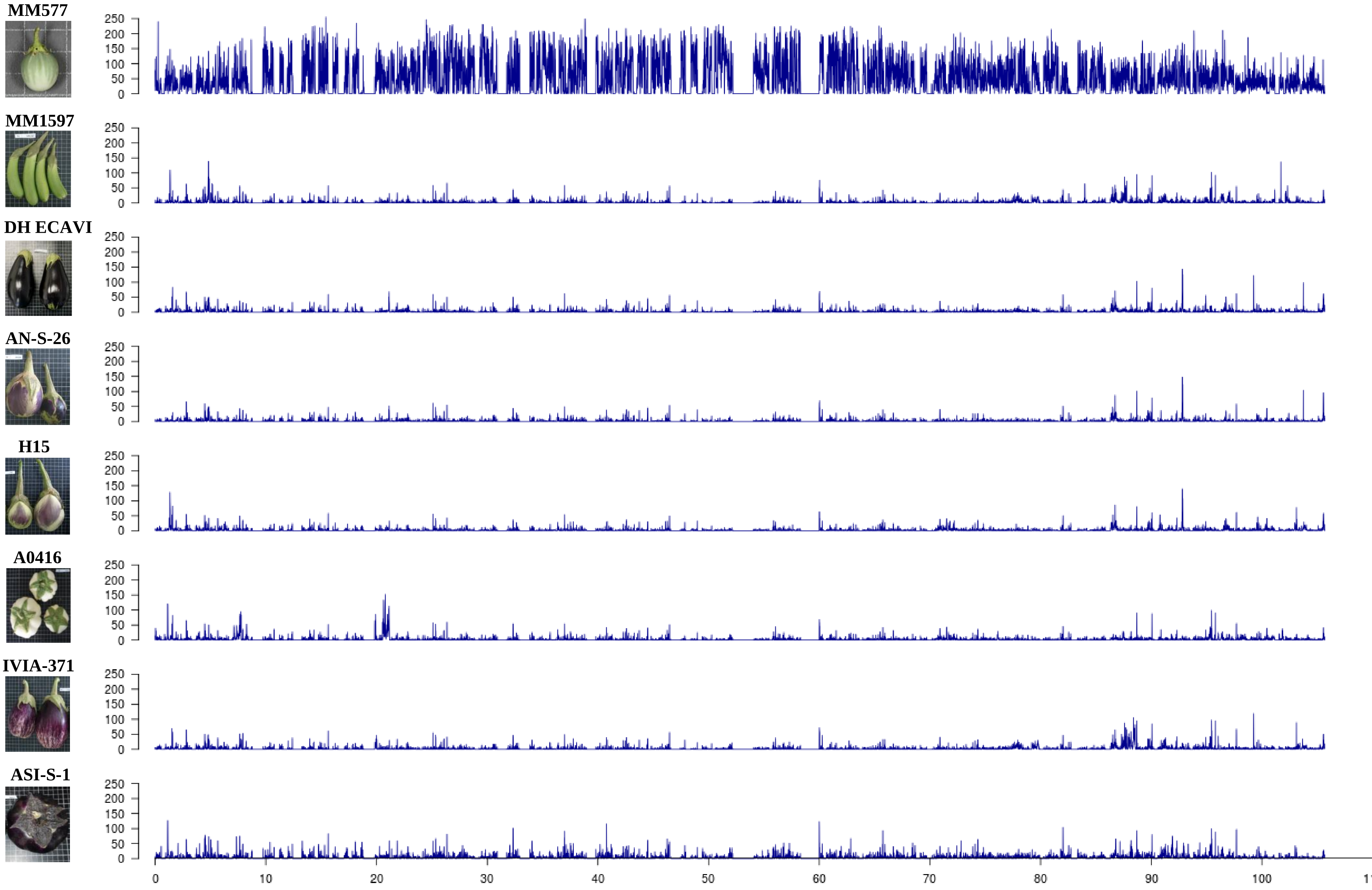

Chromosome 5 heterozygous variants

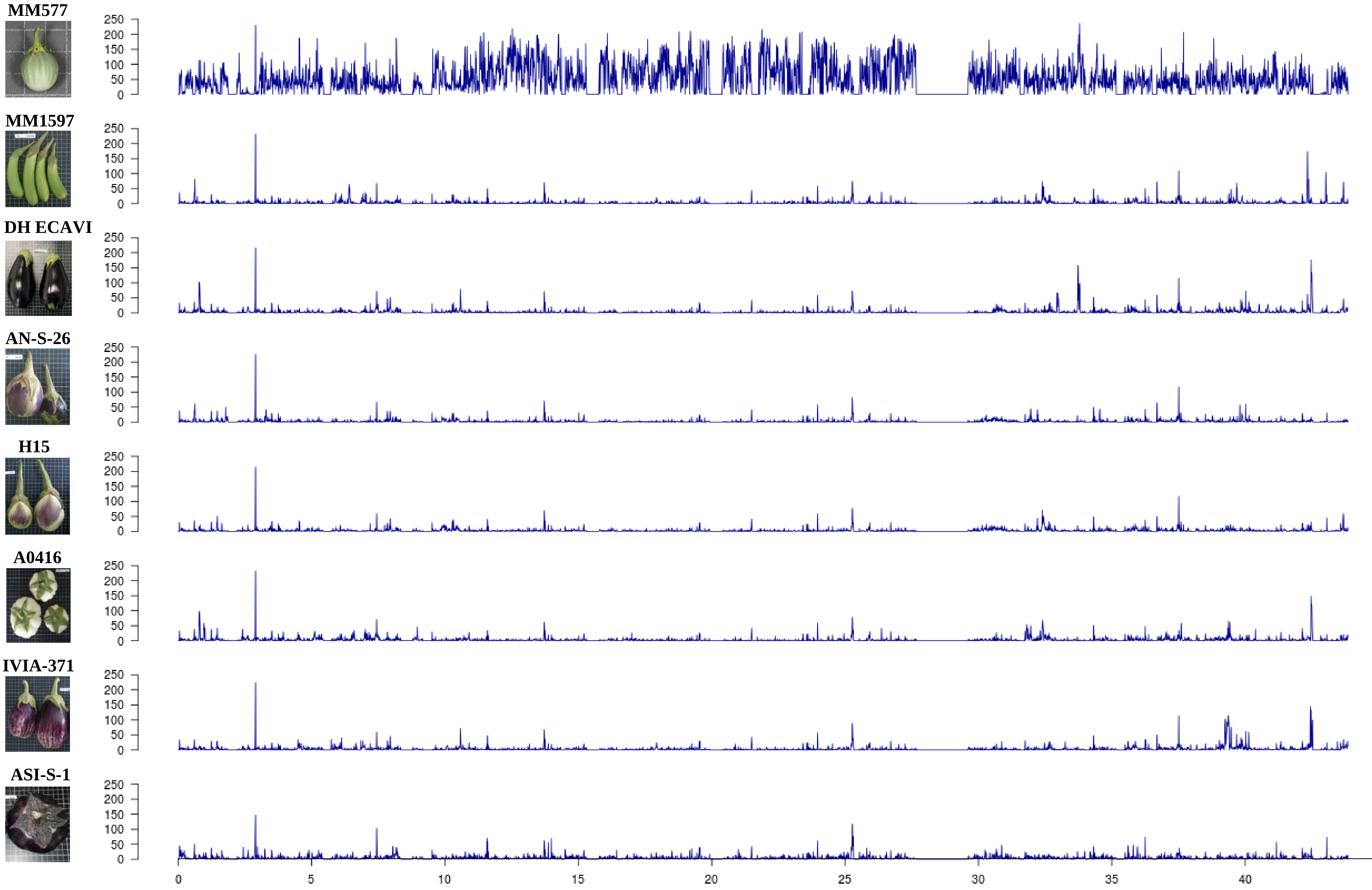

Chromosome 6 heterozygous variants

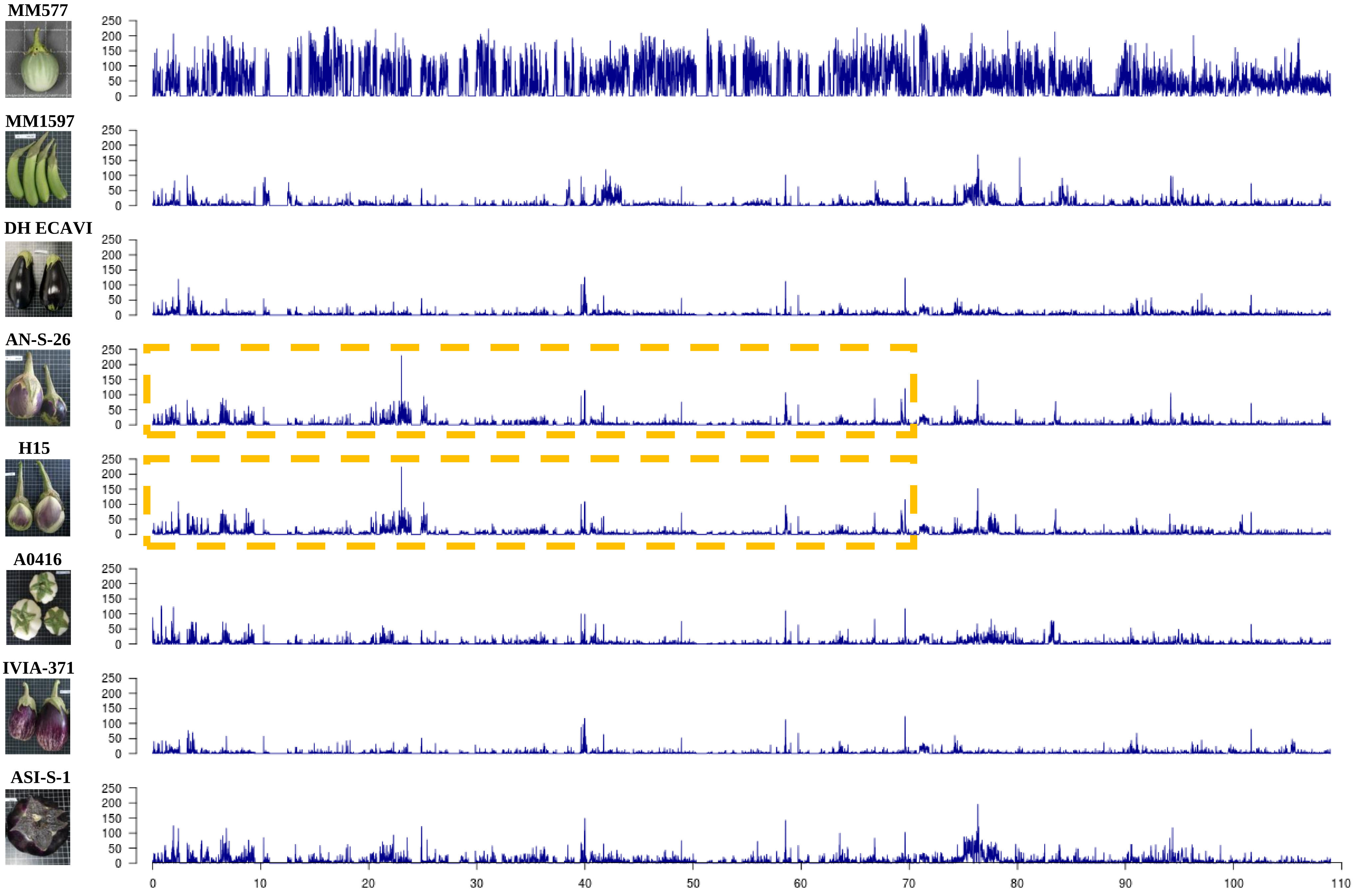

Chromosome 7 heterozygous variants

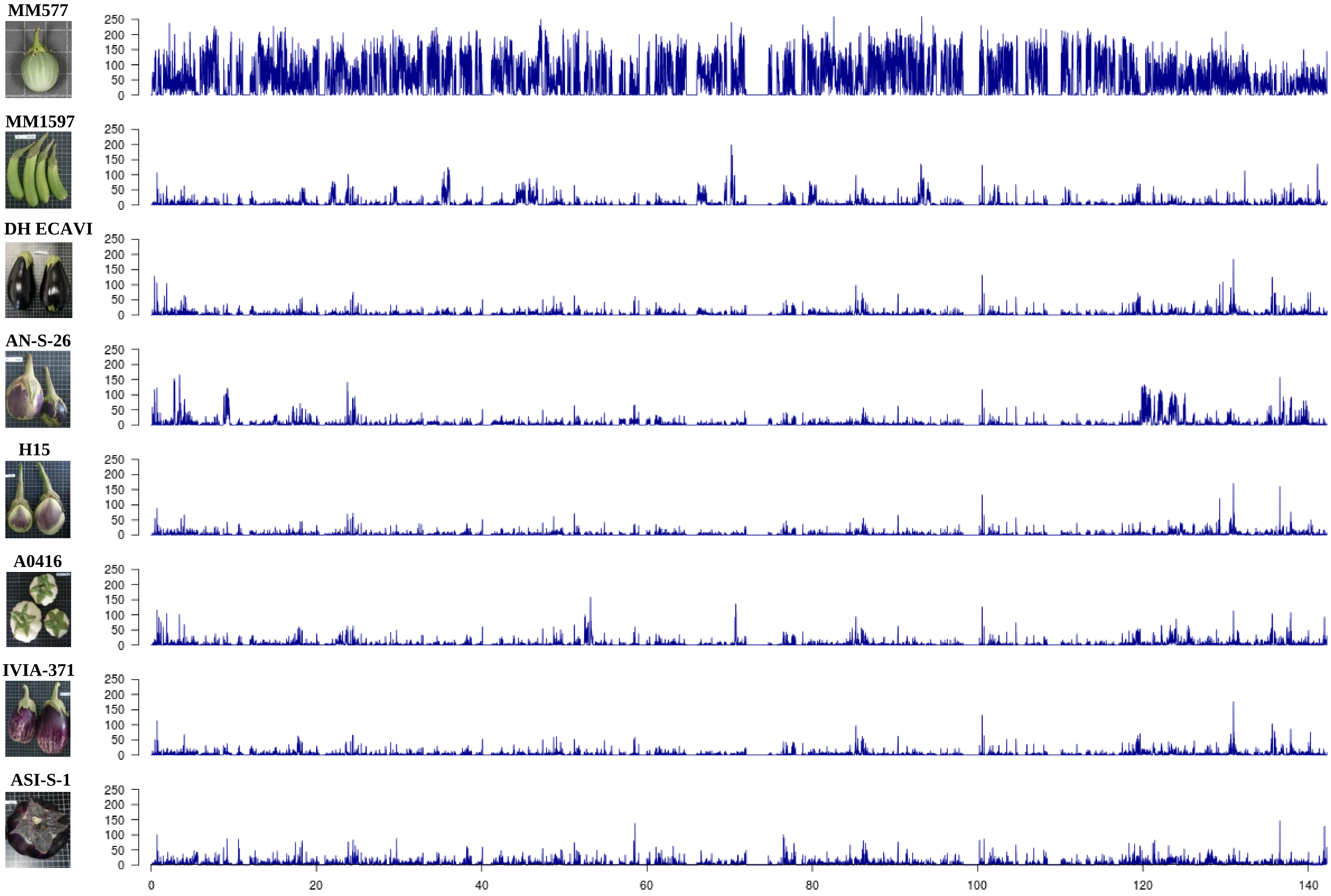

Chromosome 8 heterozygous variants

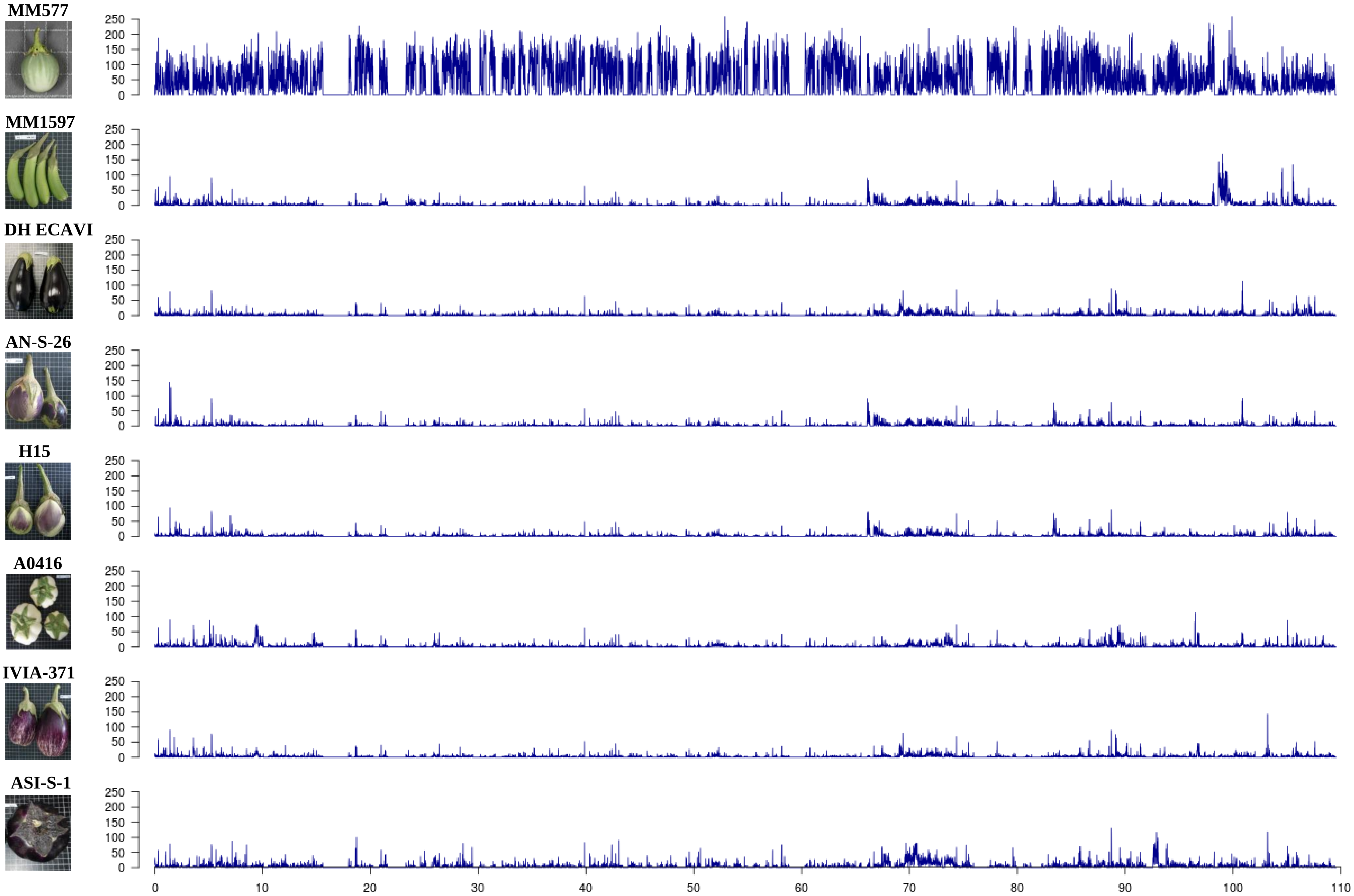

Chromosome 9 heterozygous variants

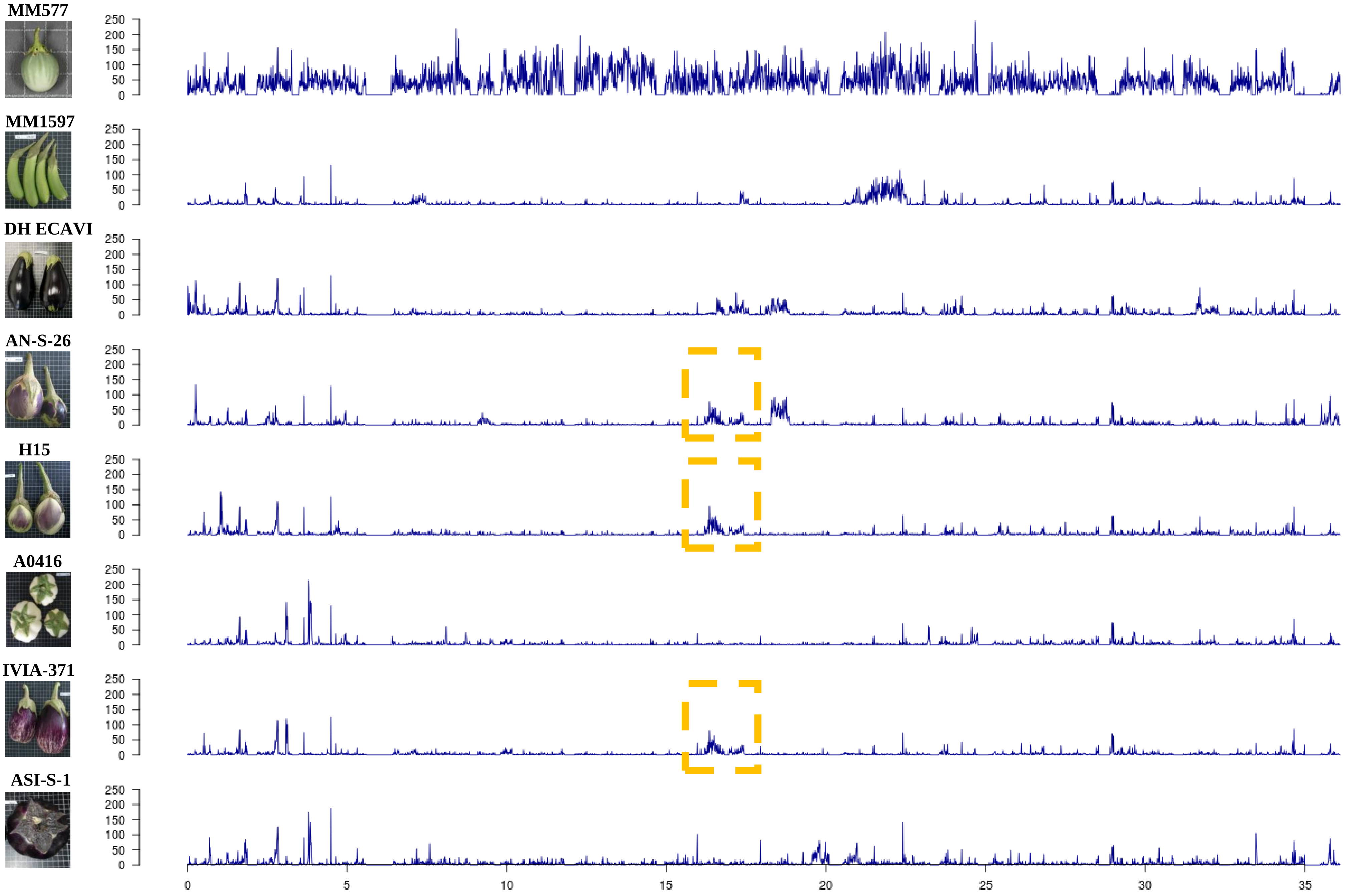

Chromosome 10 heterozygous variants

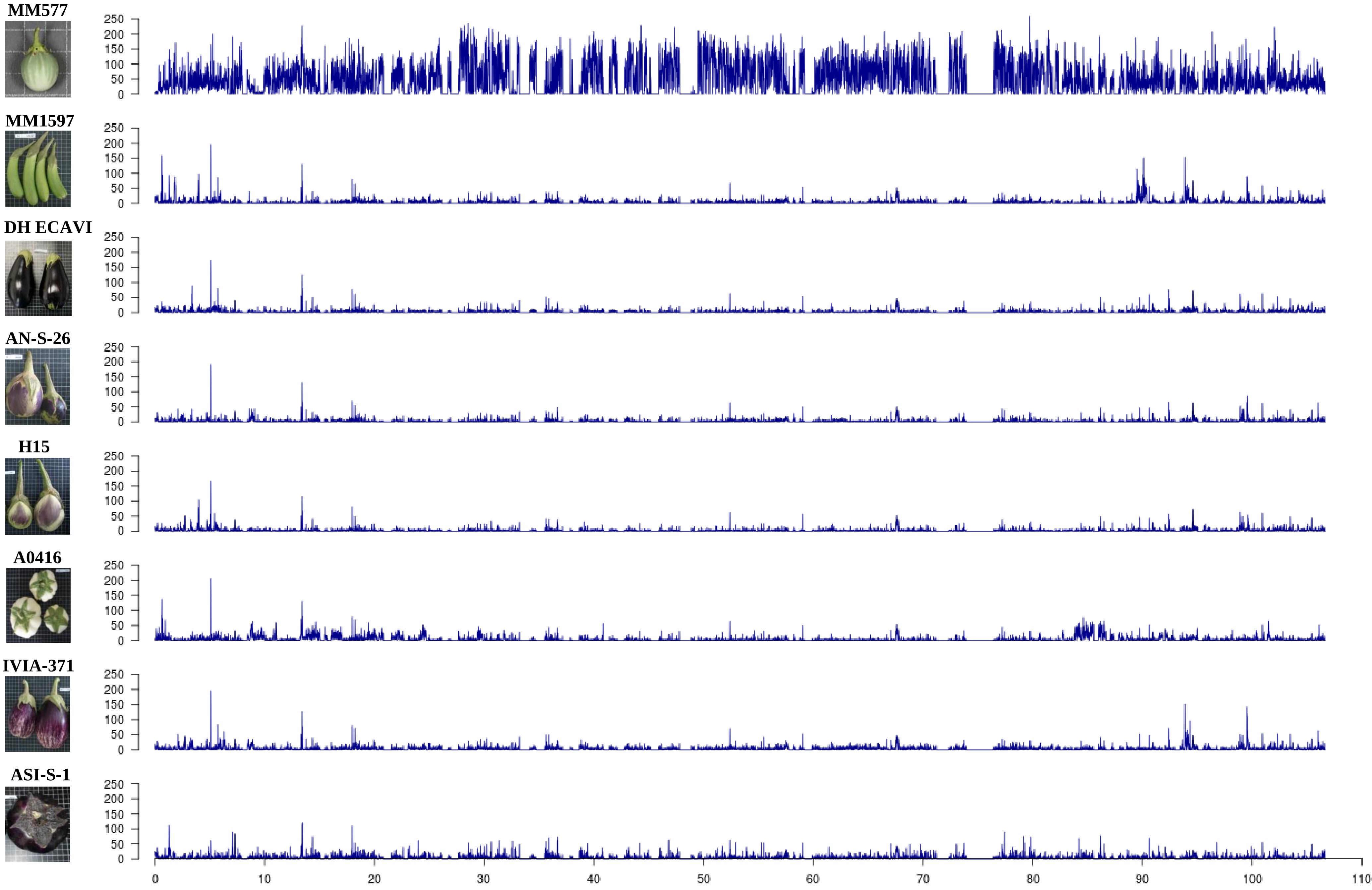

Chromosome 11 heterozygous variants

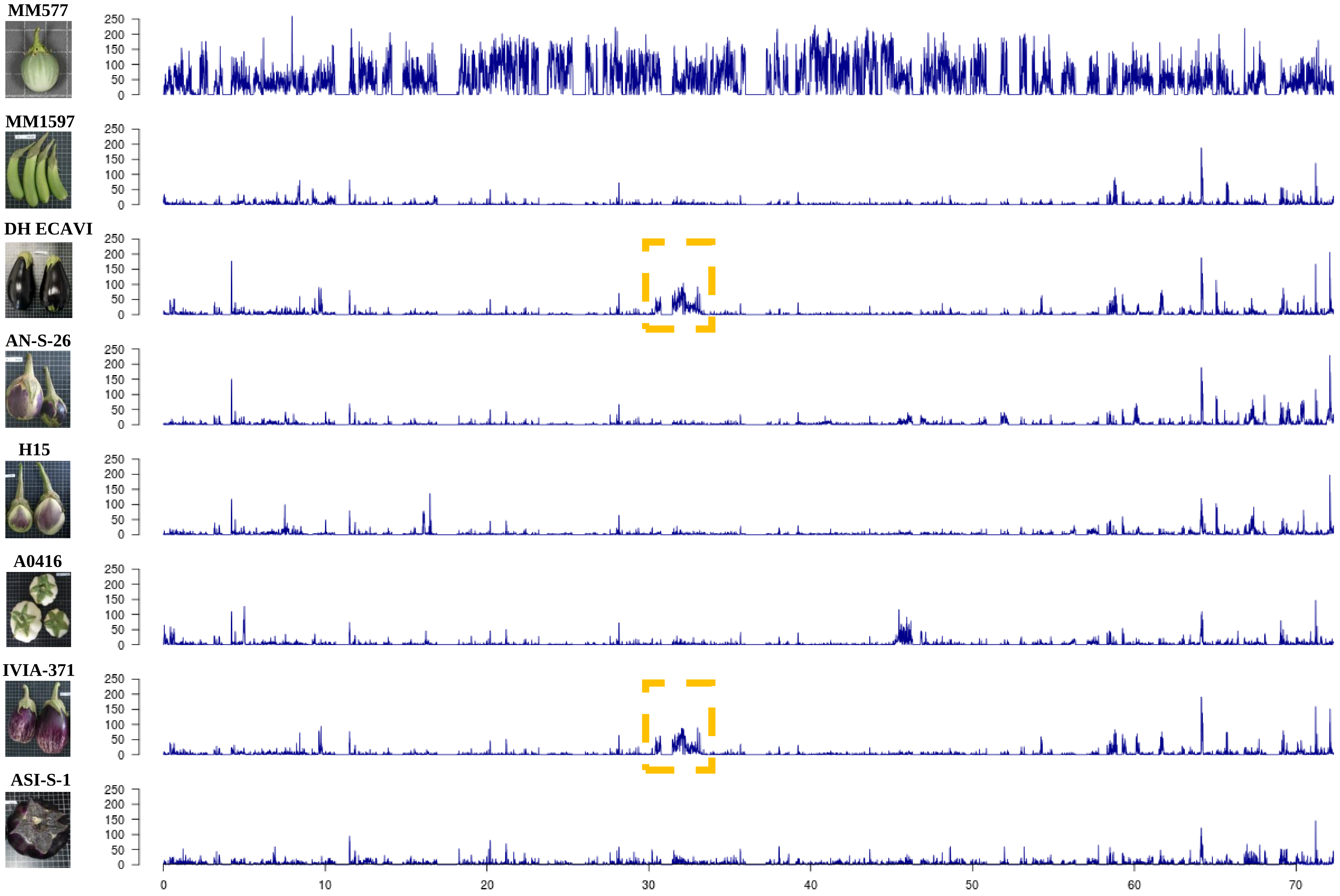

Chromosome 12 heterozygous variants

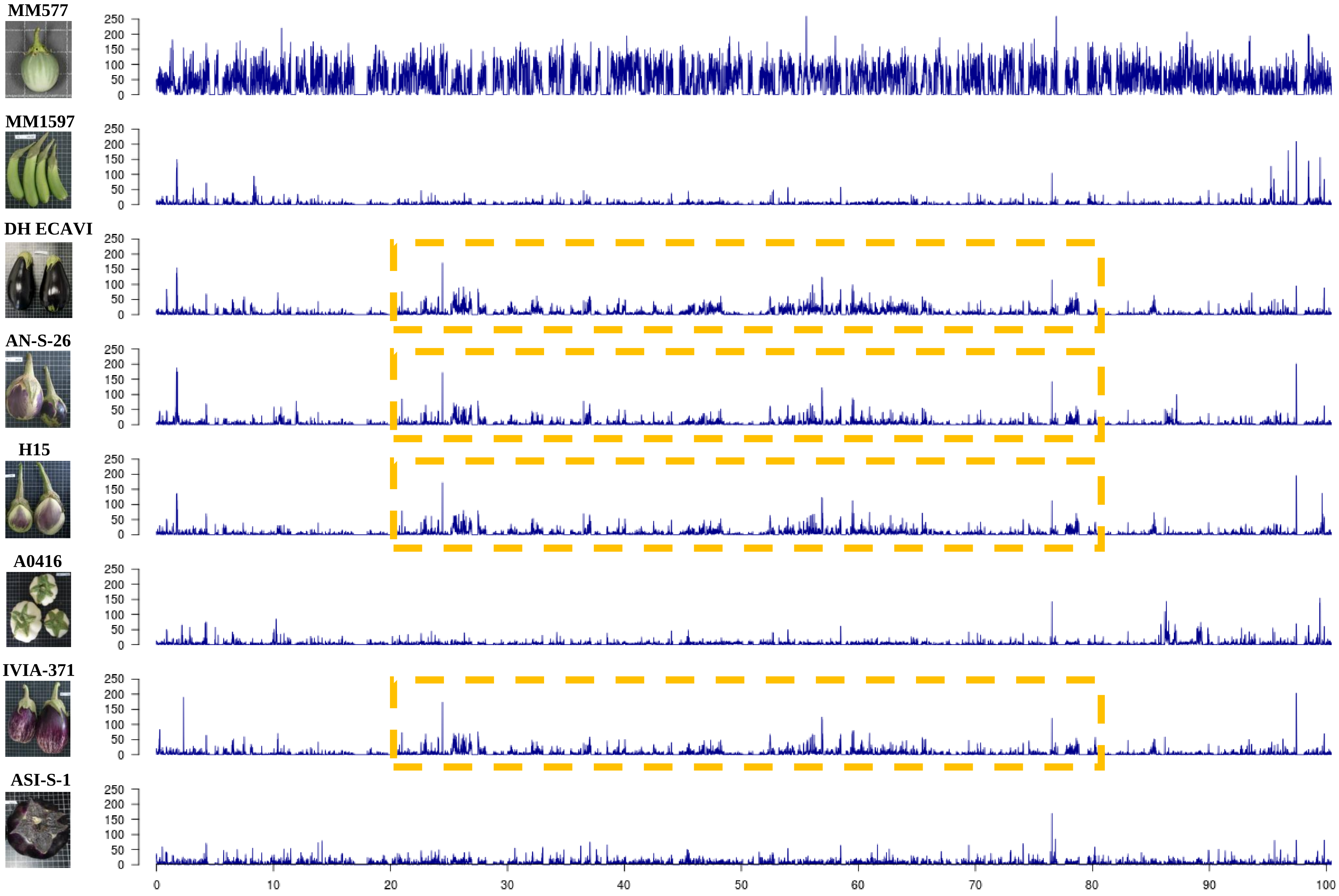

Supplement: Supplementary file 3 [file DataSheet_3.pdf]
