# Supplementary material for: Whole-Genome Resequencing of Seven Eggplant (Solanum melongena) and One Wild Relative (S. incanum) Accessions Provides New Insights and Breeding Tools for Eggplant Enhancement
Source: Front Plant Sci. 2019 Oct 8;10:1220. doi: 10.3389/fpls.2019.01220 (PMC6791922; doi:10.3389/fpls.2019.01220)
Supplement: Supplementary file 9 [file DataSheet_9.pdf]

Supplementary data S9. Transposable elements organized by class identified in the seven *S. melongena* and one *S. incanum* (MM577) accessions.

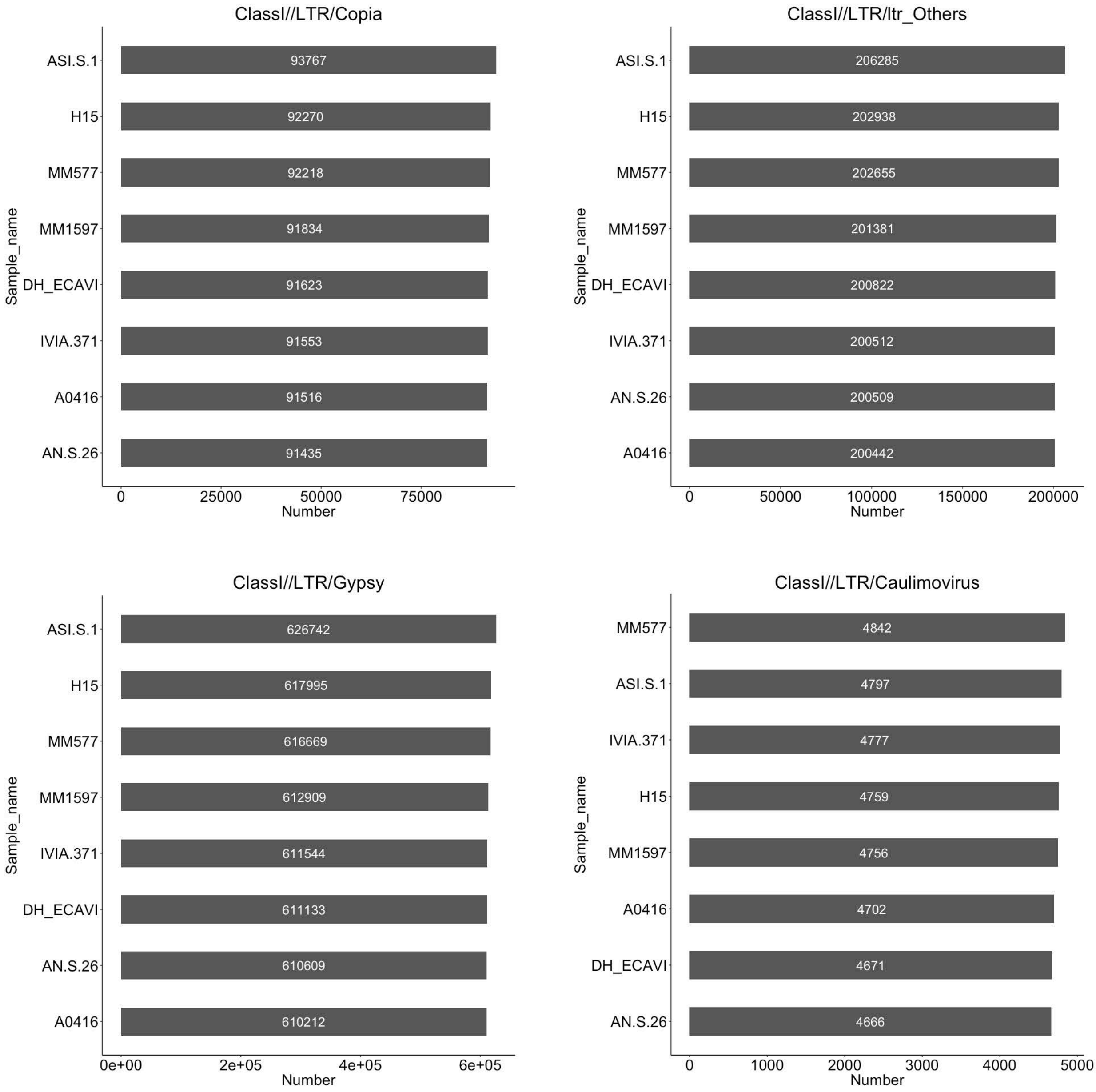

ClassI//LTR/Copia
ASI.S.1
93767
H15
92270
MM577
92218
MM1597
91834
DH_ECAVI
91623
IVIA.371
91553
A0416
91516
AN.S.26
91435
0
25000
50000
75000
Number
Sample_name
ClassI//LTR/ltr_Others
ASI.S.1
206285
H15
202938
MM577
202655
MM1597
201381
DH_ECAVI
200822
IVIA.371
200512
AN.S.26
200509
A0416
200442
0
50000
100000
150000
200000
Number
Sample_name
ClassI//LTR/Gypsy
ASI.S.1
626742
H15
617995
MM577
616669
MM1597
612909
IVIA.371
611544
DH_ECAVI
611133
AN.S.26
610609
A0416
610212
0e+00
2e+05
4e+05
6e+05
Number
Sample_name
ClassI//LTR/Caulimovirus
MM577
4842
ASI.S.1
4797
IVIA.371
4777
H15
4759
MM1597
4756
A0416
4702
DH_ECAVI
4671
AN.S.26
4666
0
1000
2000
3000
4000
5000
Number
Sample_name

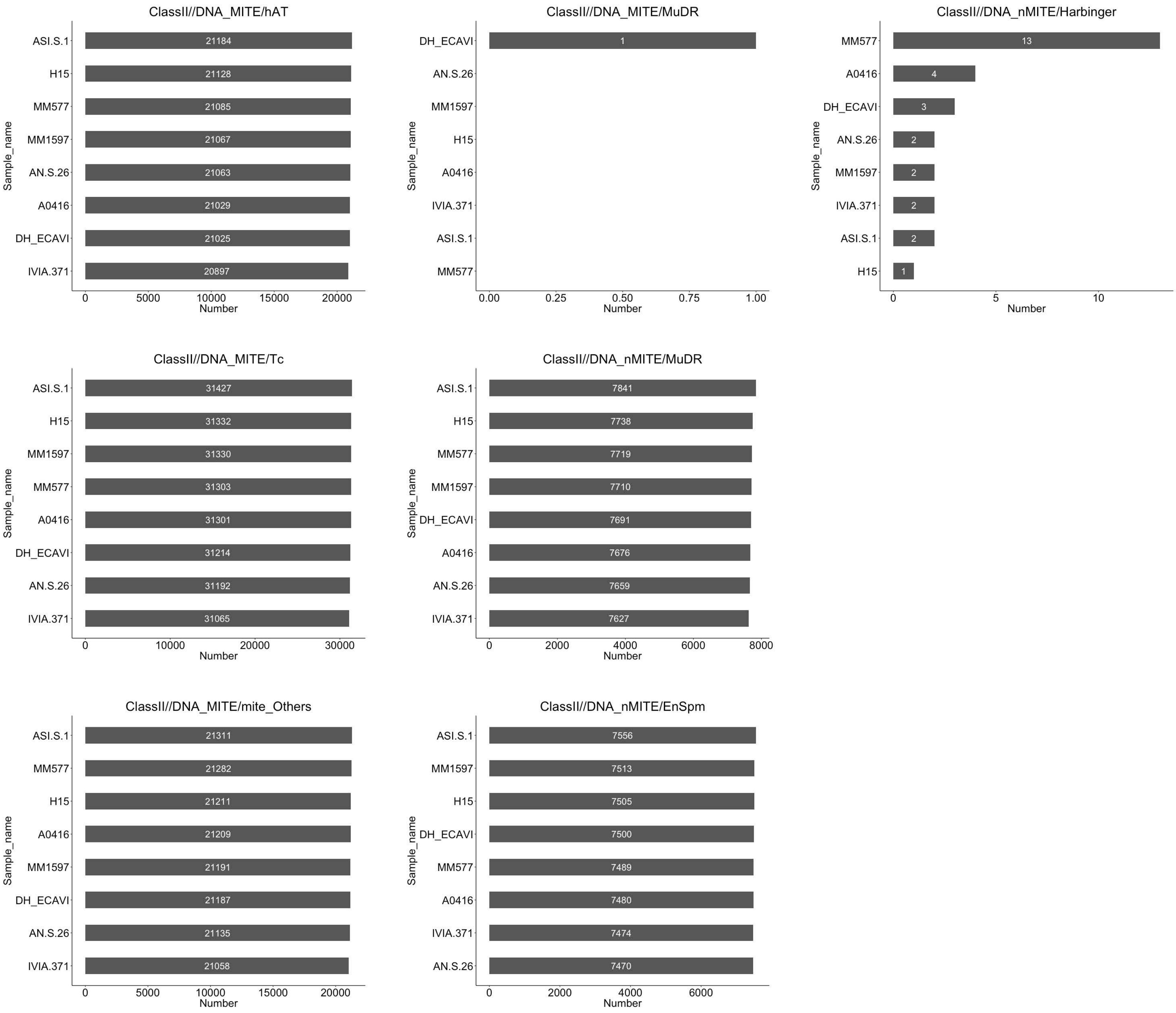
ClassII//DNA_MITE/hAT
ASI.S.1 21184
H15 21128
MM577 21085
MM1597 21067
AN.S.26 21063
A0416 21029
DH_ECAVI 21025
IVIA.371 20897
Sample_name
Number
0 5000 10000 15000 20000
ClassII//DNA_MITE/MuDR
DH_ECAVI 1
AN.S.26
MM1597
H15
A0416
IVIA.371
ASI.S.1
MM577
Sample_name
Number
0.00 0.25 0.50 0.75 1.00
ClassII//DNA_nMITE/Harbinger
MM577 13
A0416 4
DH_ECAVI 3
AN.S.26 2
MM1597 2
IVIA.371 2
ASI.S.1 2
H15 1
Sample_name
Number
0 5 10
ClassII//DNA_MITE/Tc
ASI.S.1 31427
H15 31332
MM1597 31330
MM577 31303
A0416 31301
DH_ECAVI 31214
AN.S.26 31192
IVIA.371 31065
Sample_name
Number
0 10000 20000 30000
ClassII//DNA_nMITE/MuDR
ASI.S.1 7841
H15 7738
MM577 7719
MM1597 7710
DH_ECAVI 7691
A0416 7676
AN.S.26 7659
IVIA.371 7627
Sample_name
Number
0 2000 4000 6000 8000
ClassII//DNA_MITE/mite_Others
ASI.S.1 21311
MM577 21282
H15 21211
A0416 21209
MM1597 21191
DH_ECAVI 21187
AN.S.26 21135
IVIA.371 21058
Sample_name
Number
0 5000 10000 15000 20000
ClassII//DNA_nMITE/EnSpm
ASI.S.1 7556
MM1597 7513
H15 7505
DH_ECAVI 7500
MM577 7489
A0416 7480
IVIA.371 7474
AN.S.26 7470
Sample_name
Number
0 2000 4000 6000

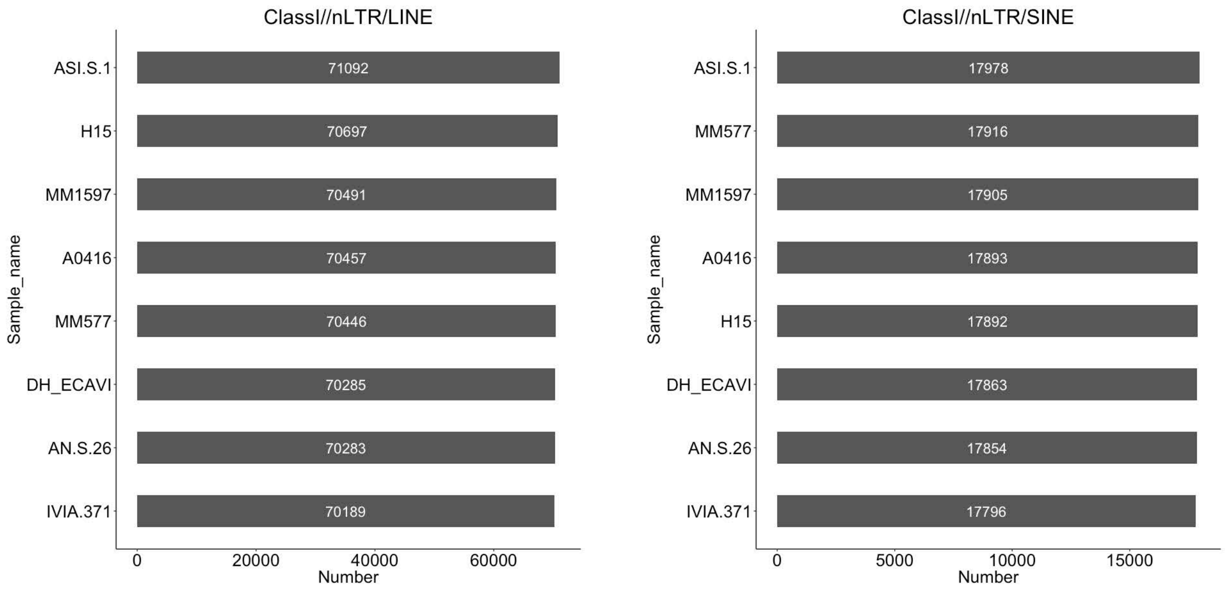
ClassI//nLTR/LINE
Sample_name
ASI.S.1
H15
MM1597
A0416
MM577
DH_ECAVI
AN.S.26
IVIA.371
71092
70697
70491
70457
70446
70285
70283
70189
0
20000
40000
60000
Number
ClassI//nLTR/SINE
Sample_name
ASI.S.1
MM577
MM1597
A0416
H15
DH_ECAVI
AN.S.26
IVIA.371
17978
17916
17905
17893
17892
17863
17854
17796
0
5000
10000
15000
Number
